# Modular, low-cost optical CT platform for hands-on tomography education

**DOI:** 10.1016/j.ohx.2026.e00852

**Published:** 2026-10-23

**Authors:** William R. Rodriguez-Dueñas, Hernan A. Bernal-Muñoz

**Affiliations:** a Department of Electronic Engineering, Faculty of Engineering, Pontificia Universidad Javeriana, Bogota, Colombia; b School of Medicine and Health Sciences, Universidad del Rosario, Bogota, Colombia

**Keywords:** Computed tomography, Educational technology, Low-cost devices, Image reconstruction, Optical tomography

## Abstract

Theoretical instruction in computed tomography frequently encounters a disconnect between abstract mathematical models and physical implementation due to the high costs and safety hazards associated with ionizing X-ray equipment. This work addresses this pedagogical barrier by developing a novel, non-ionizing, modular optical computed tomography (CT) educational platform for bioengineering teaching. Built from 3D-printed parts on a LEGO® chassis, with a Teensy 4.0 board and a visible red laser, the prototype integrates an incremental capacitive encoder that indexes each intensity sample to the carriage position. A Python-based framework implements filtered back-projection (FBP) through the inverse Radon transform, together with iterative methods such as the simultaneous algebraic reconstruction technique (SART) with Gaussian filtering or total-variation (TV) regularization. The system, realized for approximately USD 408 in its budget configuration (or USD 631 with a precision rotation stage), visualizes internal cross-sections of various phantoms, including biological analogs and refractive media. Validation trials demonstrate the efficacy of the hardware in resolving high-opacity inclusions and internal refractive structures, while using inherent acquisition artifacts as teaching examples of sampling theory and reconstruction limits. A pilot study with 16 bioengineering students showed high user acceptance (mean perception score 4.94/5) and a positive trend in conceptual understanding, providing preliminary evidence of pedagogical feasibility. By moving students from purely software-based simulation to physical hardware experimentation, this platform offers a safe, reconfigurable, and transparent tool for teaching tomographic principles. It exposes the engineering constraints, hardware limitations, and image-fidelity trade-offs that characterize medical imaging systems.

## Highlights

• Non-ionizing, modular optical CT platform for hands-on tomography education.
• Modular chassis, 3D-printed parts, Teensy 4.0, red laser, and Python reconstruction.
• A capacitive encoder synchronizes position and intensity for repeatable scans.
• Full open-source build for about USD 408, with a USD 631 precision option.
• Pilot study (n = 16) showed high acceptance and a positive learning trend.

## Graphical abstract

**Figure:**
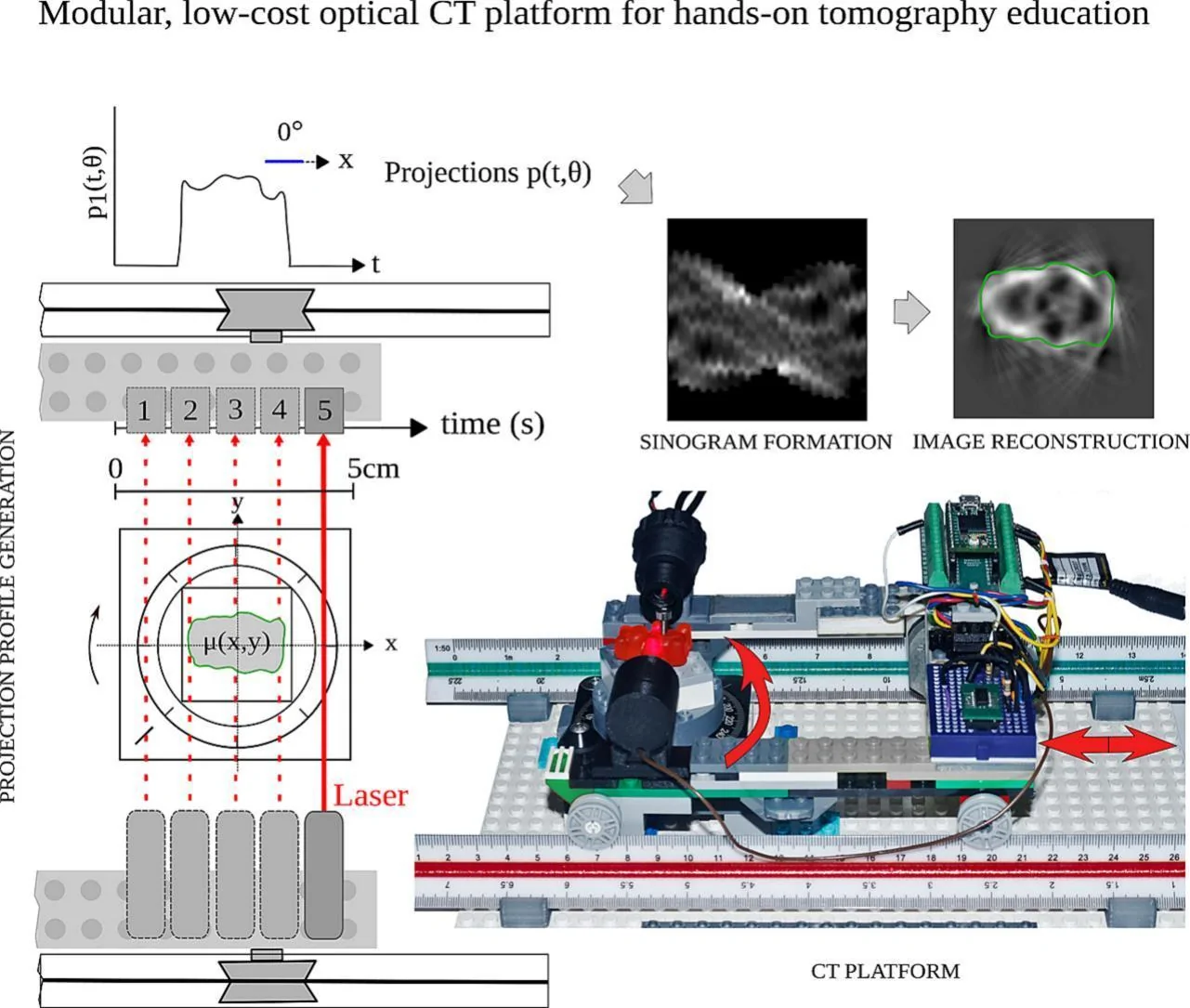

## 1 Hardware in context

Specifications Table.

**Table:** 

Hardware name	A Modular Low-Cost Optical CT Platform
Subject area	Educational tools and open-source alternatives to existing infrastructure
	Medical
Hardware type	Imaging tools
	Measuring physical properties and in-lab sensors
	Electrical engineering and computer science
Open source license	Hardware: GNU General Public License v3.0 (GNU-GPL v3.0) Software: MIT License Documentation: CC BY 4.0
Cost of hardware	Two options are provided. A2, 407.81 USD. And A1 631.14 USD. Excludes the 3D printer, soldering station, and computer.
Source file repository	https://doi.org/10.5281/zenodo.23484086

Computed Tomography (CT) is a cornerstone of modern diagnostic imaging and industrial non-destructive testing, providing a non-invasive methodology for reconstructing the internal spatial distribution of physical properties within complex specimens [1]. By mapping variables such as the linear attenuation coefficient, refractive index, or emissivity, CT hardware facilitates the visualization of internal structures that remain obscured in traditional two-dimensional radiographic projections [1], [2]. In the clinical sector, CT has fundamentally transformed diagnostics by enabling high-resolution cross-sectional analysis, allowing for the precise identification of vascular anomalies, tumors, and skeletal trauma without the superimposition of tissues [2], [3]. Beyond medicine, the relevance of CT hardware has expanded into industrial metrology and cultural heritage, where it is used for the inspection of 3D-printed medical implants and the analysis of delicate artifacts [4], [5]. Consequently, robust education in CT hardware principles is essential for medical physicists and bioengineers; however, the high cost of commercial equipment and the risks associated with ionizing radiation often limit students to software-only simulations [6], [7]. This creates a pedagogical gap, since the exclusive use of software simulation hides technical challenges and complexities inherent in acquiring real physical signals (such as mechanical misalignment, sensor noise, and temporal synchronization errors) which are fundamental to understanding the constraints of clinical image fidelity [7].

The physical architecture of a professional tomograph is defined by a sequence of hardware subsystems [8]. In the first stage, the hardware workflow begins with the generation of high-energy radiation, typically via an X-ray tube. This tube functions as the source, emitting a collimated beam that must be precisely projected through the object of interest [2], [9]. To capture the requisite multi-perspective data, the system employs a high-precision mechanical gantry. This subsystem is responsible for the synchronized x,y, and z positioning of the patient table, ensuring that the specimen remains centered within the rotation axis of the source-detector pair [6]. The mechanical stability of this positioning hardware is critical, as any submillimeter deviation can introduce significant geometric artifacts into the final image [8].

The second stage involves the physical interaction between the emitted radiation and the internal structure of the specimen. As the beam travels through the body, it undergoes attenuation based on the density of the various tissues encountered (such as bone, soft tissue, or air), each characterized by a specific linear attenuation coefficient (μ) [8]. This process is governed by the Beer-Lambert Law, where the transmitted intensity I is an exponential function of the incident intensity $I_{0}$ and the integrated density along the beam path [8]. In clinical hardware, these raw attenuation values are rescaled into Hounsfield Units, calibrated against the density of water, to provide a standardized quantitative scale for anatomical differentiation [10]. Replicating this interaction in a safe, educational environment requires a hardware setup that can accurately measure these subtle changes in intensity using non-ionizing light sources [11].

Formally, consider a parallel-beam geometry in which a ray at projection angle *θ* and detector location *t* crosses an object described by its attenuation coefficient distribution *μ*(*x*,*y*), with *s* denoting the coordinate along the ray path (Fig. 1, left). According to the Beer–Lambert law, the transmitted intensity *I* is related to the incident (reference) intensity *I_0_* by [8]:

(1) $$I\left(t,\theta \right)=I_{0}\;exp\left[-\int_{-\infty }^{\infty }\mu \left(t\;cos\theta -s\;sin\theta ,\;t\;sin\theta +s\;cos\theta \right)\;ds\right]$$

Taking the negative logarithm of the normalized intensity yields the projection *p*(*t*,*θ*) used for reconstruction:

(2) $$p\left(t,\theta \right)=-ln\left(\frac{I\left(t,\theta \right)}{I_{0}}\right)=\int_{-\infty }^{\infty }\mu \left(t\;cos\theta -s\;sin\theta ,\;t\;sin\theta +s\;cos\theta \right)\;ds$$

Eq. (2) is the Radon transform of *μ*(*x*,*y*): each projection value is the line integral of the attenuation coefficient along a ray, so that projections acquired over 0–180° in parallel-beam geometry contain sufficient information to recover the internal distribution of the object [9]. Arranged in (*t*,*θ*) space, these projections form the sinogram, in which each column corresponds to the one-dimensional profile acquired at a given angle and each point of the object traces a sinusoidal path (Fig. 1, center) [9], [10].

**Fig. 1:**
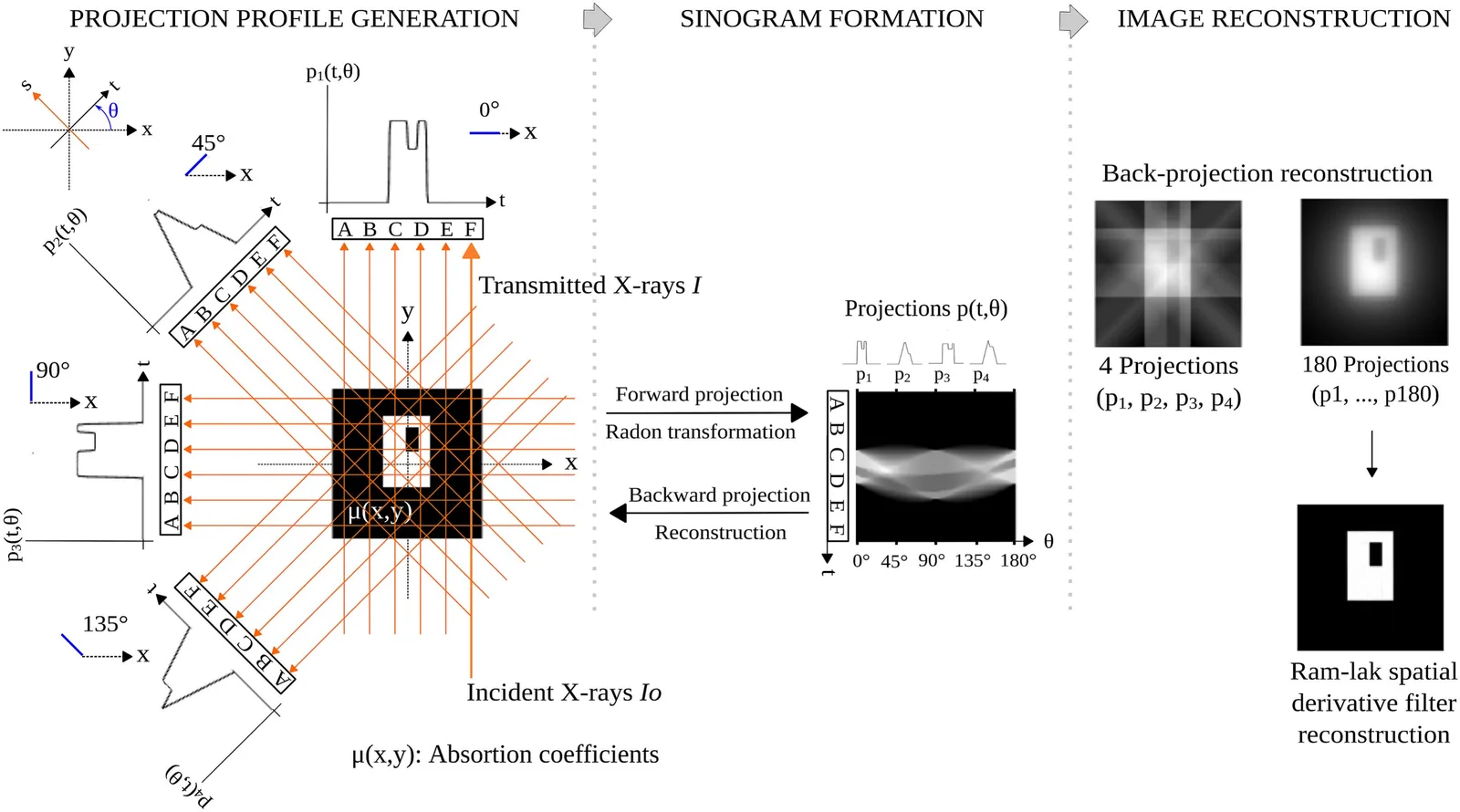
**From projection profile generation to image reconstruction in parallel-beam CT.** Left: projection profiles *p*(*t*,*θ*) obtained as line integrals of the attenuation coefficient *μ*(*x*,*y*) along the ray path *s* at detector location *t* and angle *θ* (shown for 0°, 45°, 90°, and 135°). Center: sinogram formation in (*t*,*θ*) space (forward projection, or Radon transform). Right: simple back-projection with 4 and 180 projections, and filtered back-projection with a Ram-Lak filter.

The third stage centers on the detection and digitization hardware that captures the radiation after it has exited the specimen. Modern CT detectors use solid-state scintillators that convert X-ray photons into electrical current [8]. This analog signal must be processed by a high-resolution Analog-to-Digital Converter (ADC) to prepare the data for computational analysis [8]. A primary engineering challenge in this stage is the synchronization between the mechanical position of the scanner (the angular projection) and the electrical signal acquisition. Any temporal lag between the motor movement and the sensor readout results in “smearing” within the sinogram, the visual representation of the raw data [11]. Therefore, the hardware must implement robust communication protocols (such as I2C or SPI) to ensure that each intensity measurement is correctly indexed to its corresponding spatial coordinate.

The final stage of the hardware-software workflow is image reconstruction, where the collected projection data (the sinogram) is mathematically transformed into a cross-sectional image. This is fundamentally an inverse problem solved by recovering the inner distribution of μ from a set of line integrals known as the Radon transform [1], [9]. While early systems used simple back-projection, modern hardware uses the Filtered Back-Projection algorithm, which applies convolution filters to eliminate the characteristic blurring of the back-projection process [8]. More advanced hardware-software frameworks now incorporate iterative reconstruction and algebraic techniques, which can model the physical geometry of the scanner more accurately and produce superior results even with sparse or noisy datasets [8].

Recovering *μ*(*x*,*y*) therefore amounts to inverting Eq. (2). Simple back-projection redistributes each profile uniformly along its ray paths and produces blurred images even when many projections are available (Fig. 1, right). FBP removes this blurring by convolving each projection with a ramp-type kernel (e.g., Ram-Lak or Shepp–Logan) before back-projection, an approach justified by the Fourier (central) slice theorem, which states that the one-dimensional Fourier transform of a projection acquired at angle *θ* equals the radial slice of the two-dimensional Fourier transform of the object at the same angle [3], [9]. Iterative algebraic methods (e.g., ART or SART), in contrast, refine an image estimate by comparing measured and computed projections, and are advantageous when the angular sampling is sparse [1].

Regarding physical and low-cost prototypes, researchers have demonstrated that functional micro-CT scanners can be constructed from readily available components such as portable dental X-ray tubes and intraoral digital detectors, requiring only an additional investment for precision mechanical stages [12]. Such systems, often operating at lower energies, also benefit from significantly reduced shielding requirements compared to high-energy commercial scanners, making them more amenable to classroom or small laboratory environments [5]. This approach aligns with efforts to develop cost-effective, adaptable CT systems that can be updated with evolving digital photography and imaging technologies, further enhancing their utility and longevity in educational and research settings [5].

Another category of physical prototypes involves the construction of functional, low-cost CT scanners using components or readily available real X-ray components. For instance, de Oliveira et al. demonstrated a cost-effective micro-CT system for small specimens by utilizing a low-energy X-ray tube in a fan-beam configuration with a custom-built photodiode array detector [5]. Similarly, other researchers have explored the development of optical analog CT systems, which use visible light sources and detectors instead of X-rays, providing a safe and inexpensive platform for teaching CT principles without the need for specialized radiation shielding [13]. These systems provide students with practical experience in hardware manipulation.

Despite these advancements, many educational tomography tools often consist of software-only simulations or “kitchen-based” kits [11], [14]. Although some models have used line lasers and phototransistors to emulate CT scanners [7], a persistent need remains for a platform that explicitly exposes the synchronization and digitalization stages, enabling students to troubleshoot and optimize these processes. Many existing DIY kits focus solely on the final image, often neglecting critical hardware-level challenges such as sensor calibration, position detection, and the electronic noise inherent in ADC systems.

The work presented here proposes a modular, low-cost optical CT educational platform designed to address these pedagogical and technical gaps. Our hardware follows a philosophy of total transparency and accessibility, using a building-block-based chassis and an iMXRT1062 microcontroller to provide a system that can be replicated for approximately 408 USD. The primary novelty of our work is the explicit hardware-software interface that allows a student to interact with each stage of the tomographic chain: from the physical alignment of a visible-light laser emitter to the real-time acquisition of voltage signals and their subsequent reconstruction in an open-source Python environment. Although its acquisition and reconstruction workflow resembles that of clinical X-ray CT, the platform is not a direct equivalent: it uses non-ionizing visible light, a single-detector translate–rotate (first-generation) parallel-beam geometry, and produces optical attenuation maps rather than calibrated attenuation coefficients (Section 2.3).

Unlike previous simulators, our platform incorporates an incremental capacitive encoder [15] to synchronize position and intensity during the linear translation of the specimen. This hardware-level integration indexes every intensity sample to the measured carriage position; the resulting end-to-end reproducibility of the acquisition chain is quantified in Section 7.1. By manually manipulating the hardware (specifically by adjusting the angular step, calibrating photodiode sensitivity, and observing the effects of light refraction through tissue-mimicking phantoms [13], [16]) students gain a deep, tactile understanding of the engineering compromises required in medical imaging. This “learning-by-doing” approach empowers students to move beyond the “black box” of commercial scanners, fostering a clearer understanding of the physical realities governing signal acquisition and image integrity.

The main differences between the proposed platform and other low-cost educational optical CT systems are summarized in Section 7.1. In particular, in contrast to the laser-based educational scanner of Prochazka et al. [7], which replicates a motorized third-generation geometry using 259 3D-printed parts, an FPGA controller and an array of 64 phototransistors, the present design deliberately relies on manual rotation, a single photodiode, and a dedicated incremental encoder for position–intensity synchronization, and all design files, firmware, software and datasets are openly released.

## 2 Hardware description

The proposed optical CT educational platform includes hardware and software subsystems (see Fig. 2). The hardware module consists of a platform that rotates manually 360°, and on which the specimen or object of study (in this case, a gummy bear) is placed; a four-wheeled carriage that moves on two rails and carries the radiation source (a laser module), the optical sensor, and the electronic components necessary to digitize and transmit the signal. The software module receives the digitized signals (ADC values vs position) via USB port, processes the data, and reconstructs the resulting image for display on a computer screen. These subsystems are organized into the prototype in three main stages: projection profile generation, digitization, and image reconstruction.

**Fig. 2:**
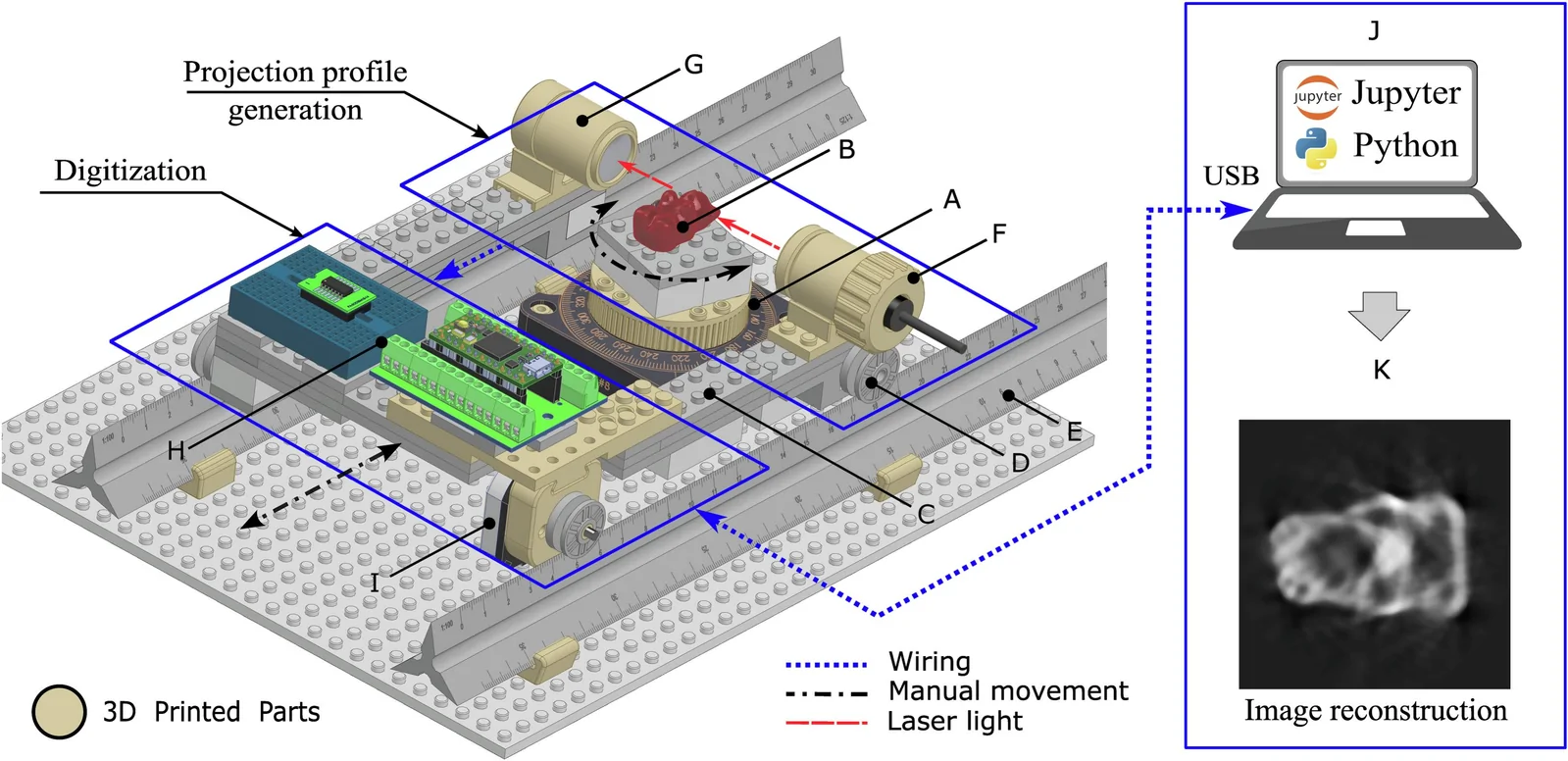
**General schematic of the system**. Platform hardware: Rotational platform (A), Object of study (B), A Carriage (C) with four wheels (D), Two scale rulers that work as rails (E), A Laser assembly (F), A sensor assembly (G), Electronic module (H), and incremental encoder assembly (I); The software module receives the digitized signals from the platform hardware via a USB port, processes the data in JupyterLab Notebook (J), and reconstructs the resultant image (K) for display on a computer screen.

The guiding principle behind the project is the development of a simple, low-cost optical CT educational platform, prioritizing accessibility and hands-on learning over high image resolution or processing speed. The main body of the system (chassis and wheels) is assembled with building blocks, chosen for their ease of assembly, reconfigurability, and widespread availability; this makes the system highly adaptable while maintaining adequate mechanical rigidity for low-speed motion. The chassis and wheels move along two rails (rulers), providing a stable guided path that supports consistent data acquisition during the projection profile generation.

### 2.1 Projection profile generation

The components for this stage are: the rotational platform, radiation source (laser module), detector sensor, and object of study (see Fig. 3). Precise angular positioning of the sample via the rotational platform is essential for acquiring projections from multiple angles, analogous to the gantry rotation in clinical CT systems and enabling accurate sinogram formation critical for reconstruction [6], [10].

**Fig. 3:**
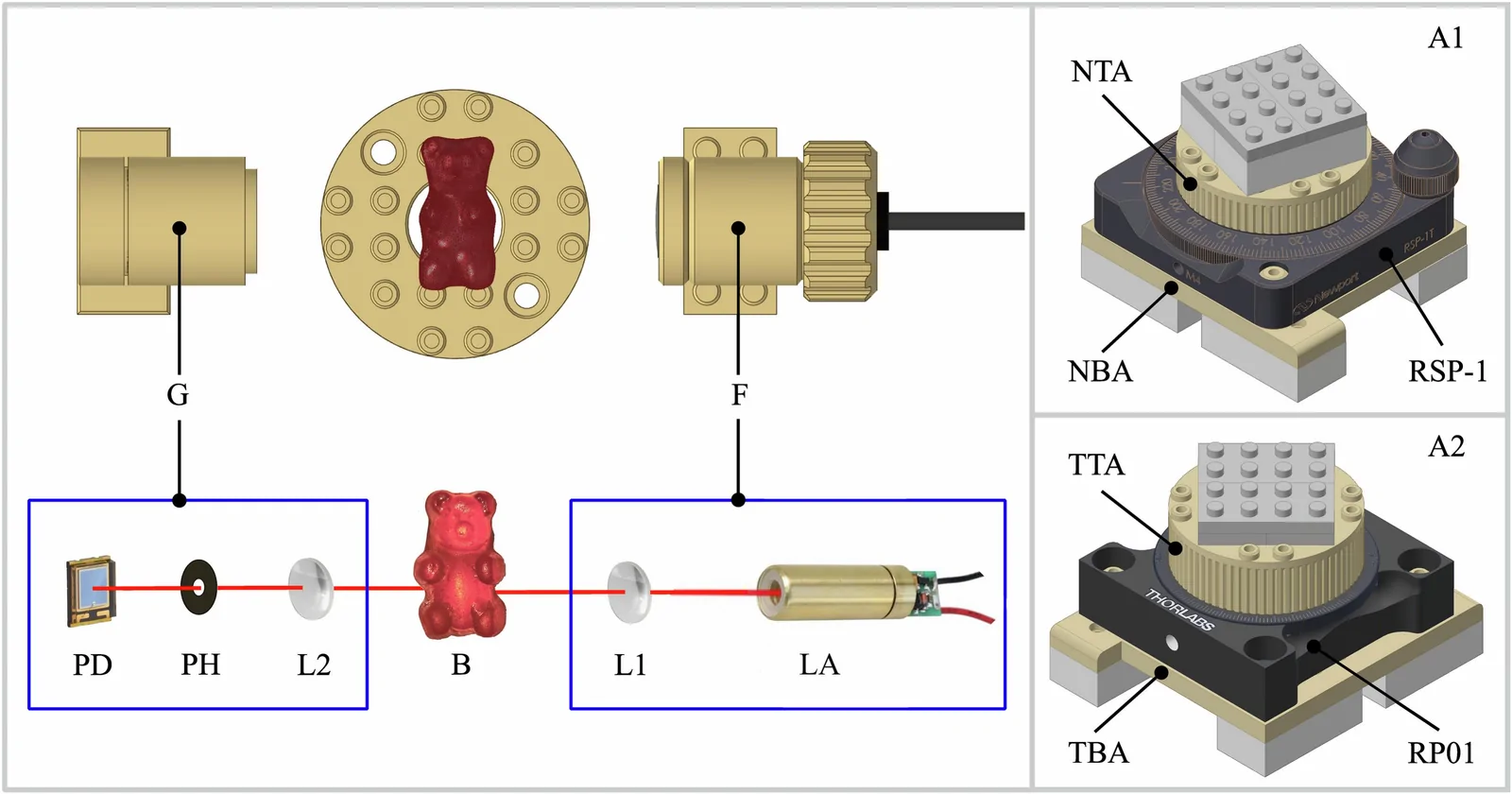
Projection profile components. Optical rotation mount (option A1: fine-adjustment knob RSP-1, NBA and NTA supports device, or A2: Standard rotational stage RP01, TBA and TTA supports), Visible laser module (LA), Object of study −gummy bear- (B), Plano-Convex Lens (L1), Double-Convex Lens (L2), Photodiode sensor (PD), And a 2–3 mm Pinhole (PH).

To align with the educational focus of the project, we prioritize low-cost and accessible components. For testing and calibration, a professional optical rotation mount (A1) such as the Newport RSP-1 T [17] was employed to ensure angular control. A more economical alternative, the Thorlabs RP01 (A2), is also included in the bill of materials [18]. These choices ensure replicability, hands-on engagement, and mechanical precision without expensive laboratory equipment, outperforming simplistic setups while maintaining pedagogical value [7].

The laser diode module (LA) acts as the energy source and provides a collimated and monochromatic light beam that approximates the pencil-beam geometry of first-generation X-ray CT, minimizing scattering and enabling well-defined projection measurements. The photodiode sensor (PD) converts the attenuated laser light into a measurable voltage signal. In front of the photodiode sensor, an *ad-hoc* 1–2 mm 3D-printed circular Pinhole (PH) can be placed to improve the acquired image; this reduces the dispersion of the light beam but reduces the signal intensity. The lenses (L1, L2) further refine the beam, with a plano-convex lens often used to focus divergent light and a double-convex lens to focus it onto the photodiode, thereby optimizing the signal-to-noise ratio and spatial resolution. This general design facilitates accessible hands-on experimentation without specialized equipment (extra 3D-printed pieces to place the laser diode, lens, and other elements can be found in the Source file repository).

The object of study (gummy bear) is positioned on the rotating platform, allowing for various cross-sectional analyses, with its translucent properties enabling effective demonstration of the principles of light attenuation (or absorption coefficient distribution μ) [14]. The gummy bear serves as a practical tissue-mimicking phantom for educational purposes, as its translucent hydrogel composition exhibits light scattering and absorption characteristics that approximate the optical behavior of soft biological tissues, making it suitable for demonstrating fundamental light-tissue interactions in tomography [16]. The setup allows for a clear visualization of how different material densities within the object affect light transmission, providing a foundational understanding of image formation in tomography [7].

### 2.2 Digitization

The system setup shown in Fig. 4 uses an iMXRT1062 microcontroller on a Teensy 4.0 board (UC) as processor unit mounted on a terminal adapter (TA), a modular incremental encoder for position feedback (I), and an operational amplifier (OP) to adapt the photodiode current for the microcontroller ADC. The Teensy 4.0 development board serves as the central processing, managing ADC data acquisition from the photodiode sensor and encoder, and communicating with the algorithms for image reconstruction. Its compact size and robust processing capabilities make it ideal for integration into the platform. Furthermore, its 12-bit analog-to-digital converter provides sufficient resolution (approximately 0.8 mV per least-significant bit) to digitize the analog signals from the photodiode sensor.

**Fig. 4:**
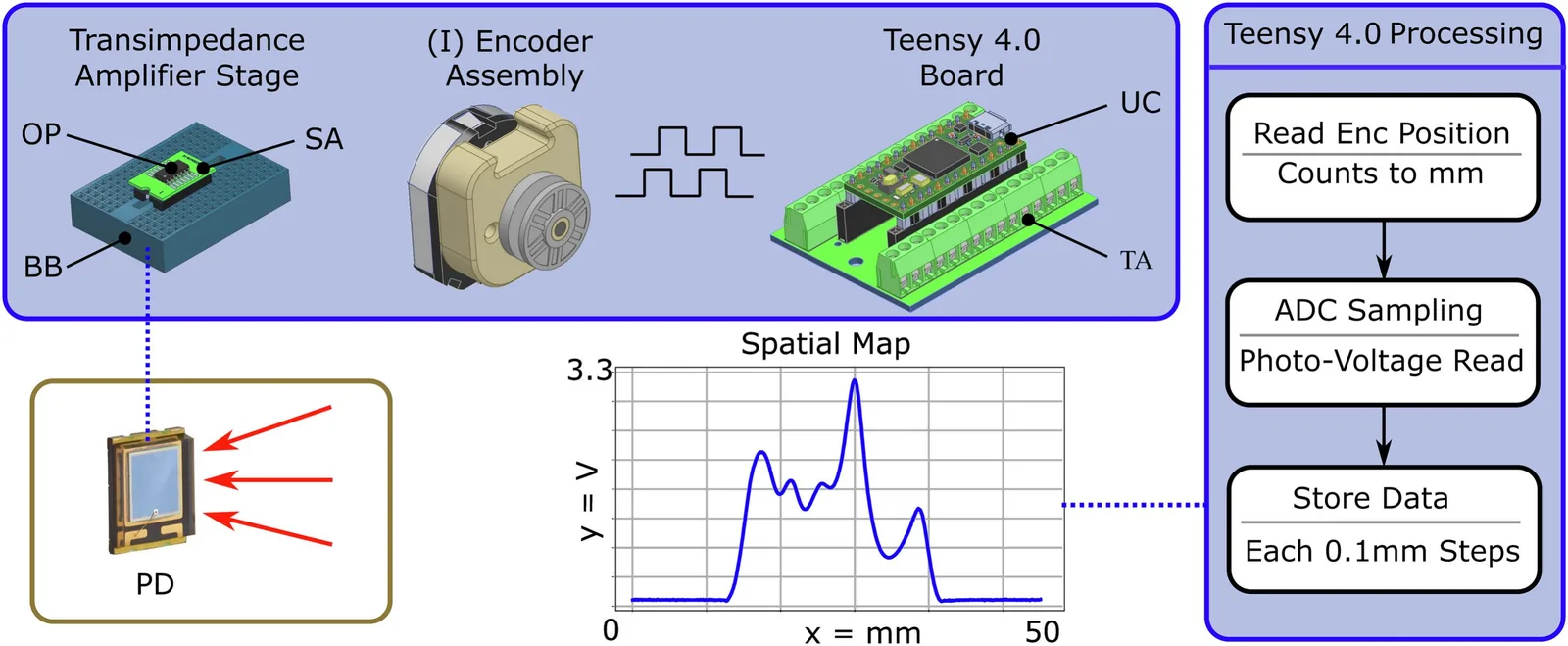
Digitization components and processing. Teensy 4.0 board (UC), Terminal adapter (TA), Operational Amplifier (OP), Soic14 to DIP adapter (SA), Breadboard (BB), and the Encoder Assembly (I) which is detailed in the build instructions section.

A key technical novelty of this low-cost architecture is the implementation of a modular incremental encoder [15] to synchronize position and intensity during the data acquisition phase. Unlike conventional DIY setups that often rely on open-loop stepper motor timing, this capacitive encoder allows the system to track the real-time linear position of the carriage with a resolution of approximately 0.012 mm per encoder count (≈86 counts per millimeter), so that each intensity measurement is indexed to its spatial position in every scan (see Section 7.1 for the experimental reproducibility assessment). This spatial indexing is essential for generating the sinogram, a fundamental requirement for tomographic reconstruction algorithms [10]. As illustrated in Fig. 5, the system executes a manual linear translation of approximately 50 mm at each angular step, of which a window of approximately 35 mm (690 samples at ≈0.051 mm) is recorded, utilizing the encoder feedback to acquire synchronized photodiode signals p(t,θ) and stores a single spatial map (projection profile) per angle; the profiles acquired at all orientations complete the sinogram of one 2D cross-sectional slice. All reconstructions reported in this work therefore correspond to 2D cross-sectional images.

**Fig. 5:**
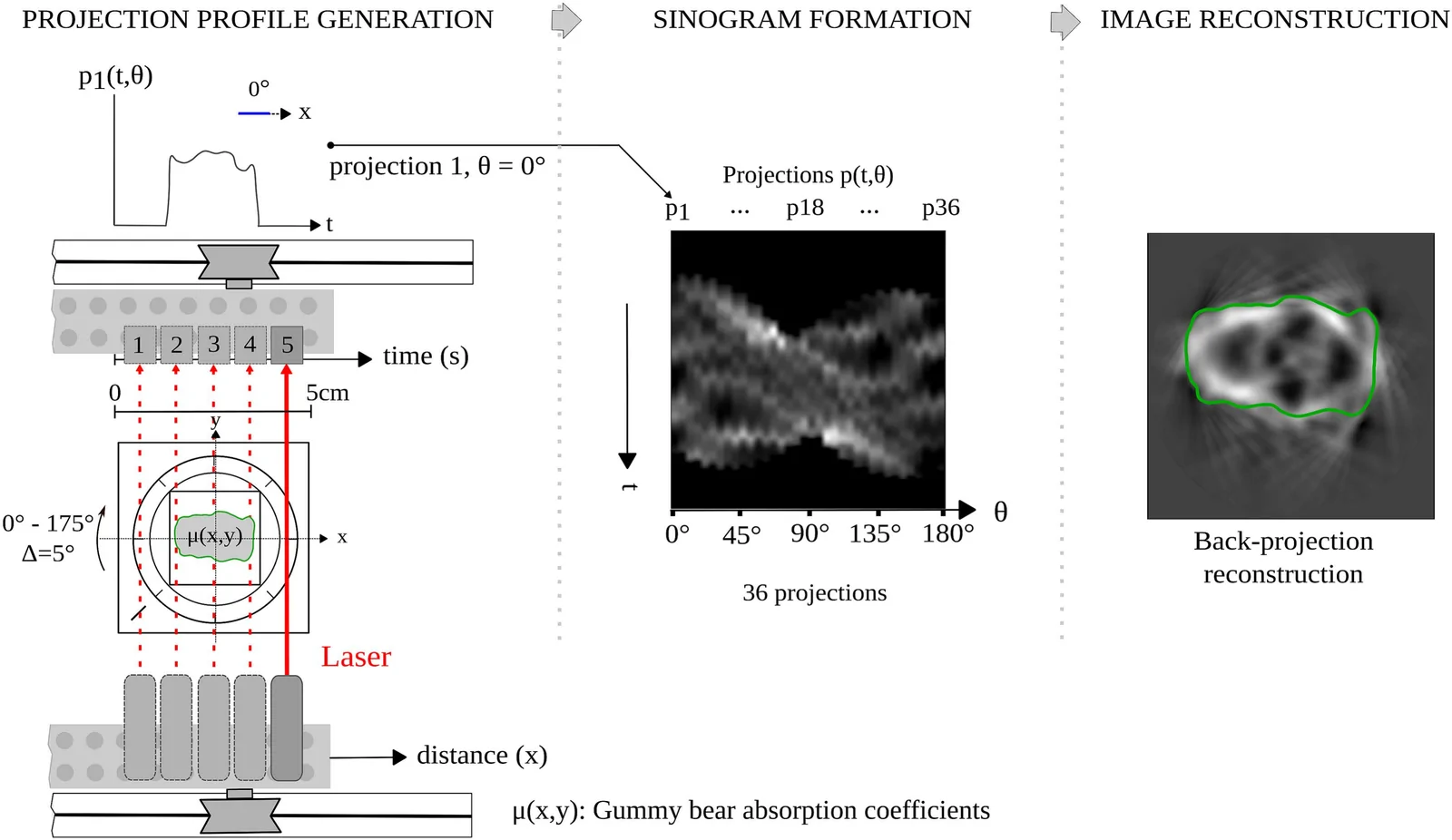
**Projection profile generation and image reconstruction.** Schematic representation of projection profile generation at angle 0° (left); sinogram formation obtained from 36 projections (center); and image reconstruction through Back-projection algorithms (right). The green outline of the object of study is superimposed on the image only as a visual reference. (For interpretation of the references to colour in this figure legend, the reader is referred to the web version of this article.)

### 2.3 Image reconstruction

This stage focuses on processing the digitized projection data to generate cross-sectional images of the scanned object (see Fig. 5). With the projection profiles obtained at each angle (p(t,θ) at angle θ and location t: p1 to p36, at 0° to 175° respectively), the system applies the reconstruction algorithms to transform these 1D profiles into a 2D image [9]. Here, the system applies a filtered back-projection method [3]. The Python algorithms used by the system include: *pyserial*, *scikit-image*, *numpy*, *matplotlib*, *time*, and *pandas* (see Source file repository for more details). This free software approach is useful in educational contexts by allowing unrestricted code reuse, facilitating interpretability and scalability without advanced computational resources [19], [20].

The digitized photodiode signal is related to the projection data through Eq. (2). The transimpedance amplifier converts the photocurrent *i*_ph_ into a voltage *V*_TIA_ = *R*_F_·*i*_ph_ (*R*_F_ = 49 kΩ), which, owing to the linear response of the photodiode, is proportional to the transmitted intensity *I*(*t*,*θ*). The firmware stores the complementary signal *S*(*t*,*θ*) = *V*_ref_ − *V*_TIA_, where *V*_ref_ = 3.3 V is the ADC full scale, so that *S* increases with attenuation. The incident (reference) intensity *I*_0_ is obtained directly from the photodiode reading of the unobstructed beam (*S*_free_ ≈ 0.03 V, i.e., *V*_TIA_ ≈ 3.27 V, corresponding to a photocurrent of approximately 60 µA), whereas a fully blocked beam yields *S*_block_ ≈ 3.27 V. The dark current of the photodiode, measured in the laboratory, was approximately 20nA (about 0.03 % of the operating photocurrent); therefore, no dark-current correction is applied. Normalizing the stored signal gives (*S* − *S*_free_)/(*V*_ref_ − *S*_free_) = 1 − *I*/*I*_0_, which is the first-order approximation of − ln(*I*/*I*_0_) in Eq. (2) for weak attenuation. For this reason, the logarithmic transformation was not applied to the results reported in this work, and the digitized profiles were used directly as input to the reconstruction algorithms. This approximation preserves the location and shape of internal features, which is the primary pedagogical objective, but it compresses the dynamic range of strongly attenuating regions, where 1 − *I*/*I*_0_ saturates at unity while − ln(*I*/*I*_0_) increases without bound; consequently, the reconstructed gray levels should not be interpreted as quantitative attenuation coefficients.

Because the firmware bins the encoder-indexed samples at fixed intervals of approximately 0.051 mm (verified with phantoms of known diameter; Section 7.1), each projection comprises 690 samples covering a field of view of approximately 35 mm, and the reconstructed images inherit this sampling, yielding a native pixel size of approximately 0.051 × 0.051 mm (≈0.0026 mm^2^ per pixel). This value describes the reconstruction grid rather than the effective spatial resolution, which is limited primarily by the width of the laser beam and the pinhole aperture placed in front of the photodiode (Section 2.1).

Potential applications for other researchers.

● Teaching laboratories: instructors in biomedical engineering, physics, and medical imaging can use the platform to demonstrate the complete CT workflow (projection acquisition, sinogram formation, and image reconstruction) with real data and without ionizing radiation.
● Reconstruction and image processing: the acquired sinograms can serve as real, test data for comparing reconstruction algorithms, such as FBP and iterative methods, under different angular sampling conditions.
● Optical measurements: the encoder-indexed transmission scan can be used to obtain one-dimensional intensity profiles of translucent or opaque samples, a common task in basic optics and instrumentation practicals.
● Low-cost instrumentation development: the open design files, firmware, and software can serve as a starting point for adapting the scanner to other light sources, detectors, or motorized stages.

## 3 Design files summary

The structural and mechanical components of this platform were designed using Autodesk® Inventor® Professional 2023. To maximize accessibility and comply with open-source hardware practices, the repository includes the original CAD files for all custom-designed components in both their native Autodesk Inventor formats (.ipt,.iam) and the widely supported STEP (.step) exchange format. No third-party CAD geometries are distributed with the repository (see Table 1 for more information).

**Table 1:** Summary of design, software and fabrication files available in the source file repository.

**Design filename**	**File type**	**Open source license**	**Location of the file**
Original design files	Autodesk® parts and assemblies.IPT,.IAM files	GNU-GPL v3.0	\hardware\ CAD_files\source\
CAD data exchange	ISO 10,303.STEP files	GNU-GPL v3.0	\hardware\CAD_files\export\
Fabrication files	3D model.STL	GNU-GPL v3.0	\hardware\Fab_files\
3D Printing Guide	.PDF Document	CC BY 4.0	\hardware\
Bill_of_materials	Spreadsheet.XLSX	CC BY 4.0	\hardware\BOM\
CT_Reconstruction	JupyterLab Notebook.IPYNB file	MIT License	\software\
Tomo_firmware.ino	Arduino(IDE).INO	MIT License	\firmware\Tomo_firmware\
Notebook_Usage_Guide	.PDF Document	CC BY 4.0	\software\docs\
Spatial_Maps_Dataset	.CSV files	CC BY 4.0	\software\data\
Expected_Results	.PNG Image files	CC BY 4.0	\software\data\samples\

To facilitate fabrication, a comprehensive graphical 3D printing guide is provided, detailing the recommended printing parameters for each custom component. Assembly documentation includes rendered images illustrating the complete system configuration, while avoiding redistribution of proprietary third-party CAD models.

To enable users to evaluate the software pipeline independently of the hardware, the repository also provides a standalone Jupyter Notebook user guide, complete spatial map datasets (.csv), and representative sample output images.

## 4 Bill of materials summary

The Bill of Materials (Table 2) is quoted in U.S. dollars and can be sourced from different suppliers. The most expensive component, the rotational stage, has two alternative configurations allowing for different budget levels. These are listed separately in Table 3 (both options and its 3D-printed support adapters are included in the design files summary). The reported totals include all components listed in Table 2 (electronic, optical, and mechanical parts, LEGO® elements, and 3D-printing filament). General-purpose equipment (the 3D printer, soldering station, and computer) is not included in the cost analysis, since it is commonly available in teaching laboratories.

**Table 2:** Bill of materials. Parts prices and links accessed September 2026.

**ID**	**Qty**	**Part code**	**Unit cost (USD)**	**Extended cost (USD)**	**Description**	**Link**
B1	1	SUTB693ZZ	13.77	13.77	Ball bearing. 3 mm Shaft, Housing 8 mm, Width 4 mm	https://us.misumi-ec.com/
B2	1	FL623ZZ	9.14	9.14	Deep Groove Ball Bearing − Flanged	https://us.misumi-ec.com/
SH	1	SSFHR3-40	7.74	7.74	3 mm Dia × 40 mm L Rotary Shaft − Straight	https://us.misumi-ec.com/
EN	1	102-1308-ND	28.25	28.25	AMT103-V ENCODER PROG SW TTL AXIAL	https://www.digikey.com/
EC	1	102-1504-ND	6.41	6.41	Encoder Cable ASSY 5X22AWG 1′	https://www.digikey.com/
PD	1	PDB-C171SMC	4.49	4.49	PHOTODIODE PIN	https://www.digikey.com/
L2	1	#63-538	33.25	33.25	Lens 25 mm fl, 10 mm Dia	https://www.edmundoptics.com/
L1	1	#37-793	31.70	31.70	Lens 45 mm fl, 12.5 mm Dia	https://www.edmundoptics.com/
LA	1	VLM-635-60 LPT	63.84	63.84	Laser module 635 nm 1mW 10 mm Dia	https://www.digikey.com/
PS	1	237-2255-ND	7.54	7.54	5 V 10 W AC/DC External (Class II) Adapter	https://www.digikey.com/
BJ	1	CP-024B-ND	5.17	5.17	CONN PWR JACK 2.5X5.5MM SOLDER	https://www.digikey.com/
R1,R2	2	CFR-25JB-52-1 K	0.10	0.20	RES 1 K OHM 5 % 1/4W AXIAL	https://www.digikey.com/
R3,R4	2	CFR-25JB-52-2 K	0.10	0.20	RES 2 K OHM 5 % 1/4W AXIAL	https://www.digikey.com/
R5	1	MFR-25FRF52-1 M	0.10	0.10	RES 1 M OHM 1 % 1/4W AXIAL	https://www.digikey.com/
RF	1	MF1/4DCT52R4992F	0.10	0.10	RES 49.9 K OHM 1 % 1/4W AXIAL	https://www.digikey.com/
C1	1	K101J10C0GF5UH5	0.22	0.22	CAP CER 100PF 50 V C0G/NP0 RADIAL	https://www.digikey.com/
SA	1	PA0003-ND	3.49	3.49	SOIC-14 TO DIP-14 SMT ADAPTER	https://www.digikey.com/
UC	1	1568-16997-ND	26.80	26.80	iMXRT1062 Teensy 4.0 ARM® Cortex®-M7 MCU	https://www.digikey.com/
TA	1	B07CBSMQ6T	3.50	3.50	Terminal Adapter IO Shield for Nano	https://www.amazon.com/
OP	1	296-35336-1-ND − Cut Tape (CT)	3.65	3.65	LMH6644 Opam	https://www.digikey.com/
BB	1	2183-1488-ND	4.95	4.95	Solderless Breadboard (47.0 mm × 35.0 mm)	https://www.digikey.com/
E	2	B0F2S7MBY2	5.00	10.00	Architectural Scale Ruler, 12″ Plastic	https://www.amazon.com/
C,D	53	\hardware\BOM	0.43	22.87	Lego Bricks, different sizes	https://www.lego.com/
–	2	Wire 28AWG 20 cm	0.00	0.00	Photodiode wire (from Stripped USB cable)	
A,F,G,I	19	Multiple references	0.06	1.12	PLA Black Filament, 80 g required	\hardware\Fab_files\

**Table 3:** Rotation stage option A1 or A2 bill of materials. Accessed September 2026.

**ID**	**Qty**	**Part code**	**Unit cost (USD)**	**Extended cost (USD)**	**Description**	**Link**
A1	1	RSP-1	342.00	342.00	Optic Rotation Mount, 25.4 mm, Fine Adjust Knob	https://www.newport.com/
A2	1	RP01	119.18	119.18	Ø2″ Manual Rotation Stage	https://www.thorlabs.com/
A1	2	K0869.04X10	0.32	0.64	Hex Socket Head Screws M4 × 10	https://us.misumi-ec.com/
A2	1	25C62KCS	0.13	0.13	Hex Socket Cap Screw 1/4″ 5/8″L	https://us.misumi-ec.com/

Rotational stage option:

• Budget option total (USD 407.81): requires part A2 (see Table 3).
• Premium option total (USD 631.14): requires part A1 (see Table 3).

Summary of the 104 components used by type:

• 21 3D-printed adapter and support parts.
• 53 Standard LEGO® parts.
• 30 Mechanical and electronic components from different manufacturers.

Tools used (not included in the cost analysis):

• Soldering station Hako®.
• 3D printer (Ultimaker®).
• Hex Key 3/16″ for the Thorlabs rotation stage Option (A2).
• Personal computer (Windows or Linux) running JupyterLab for data acquisition and image reconstruction.
• Hex Key 3 mm for the Newport stage Option (A1).

## 5 Build instructions

The assembly of the modular optical CT platform is divided into three main stages: mechanical frame construction, electronic circuit integration, and optical alignment. All 3D-printed components referenced in this section are available in the Design Files summary at the Zenodo repository of the project.

### 5.1 Mechanical assembly

The mechanical structure uses a building-block architecture to provide a balance between modularity and cost-effectiveness while maintaining sufficient structural rigidity for low-speed motion. The final shape and dimensions of the U-shaped linear carriage can be customized based on the user's available LEGO pieces; the design presented here measures 120 mm width and 152 mm length. The list and references for the LEGO components used, along with the design files for the necessary custom 3D-printed parts, are available in the hardware section of the project repository.

1. Frame construction: Assemble the U-shaped carriage structure using standard LEGO bricks as shown in Fig. 6. This U-shaped geometry is designed to allow the laser to scan the phantom without obstruction as it rotates.

**Fig. 6:**
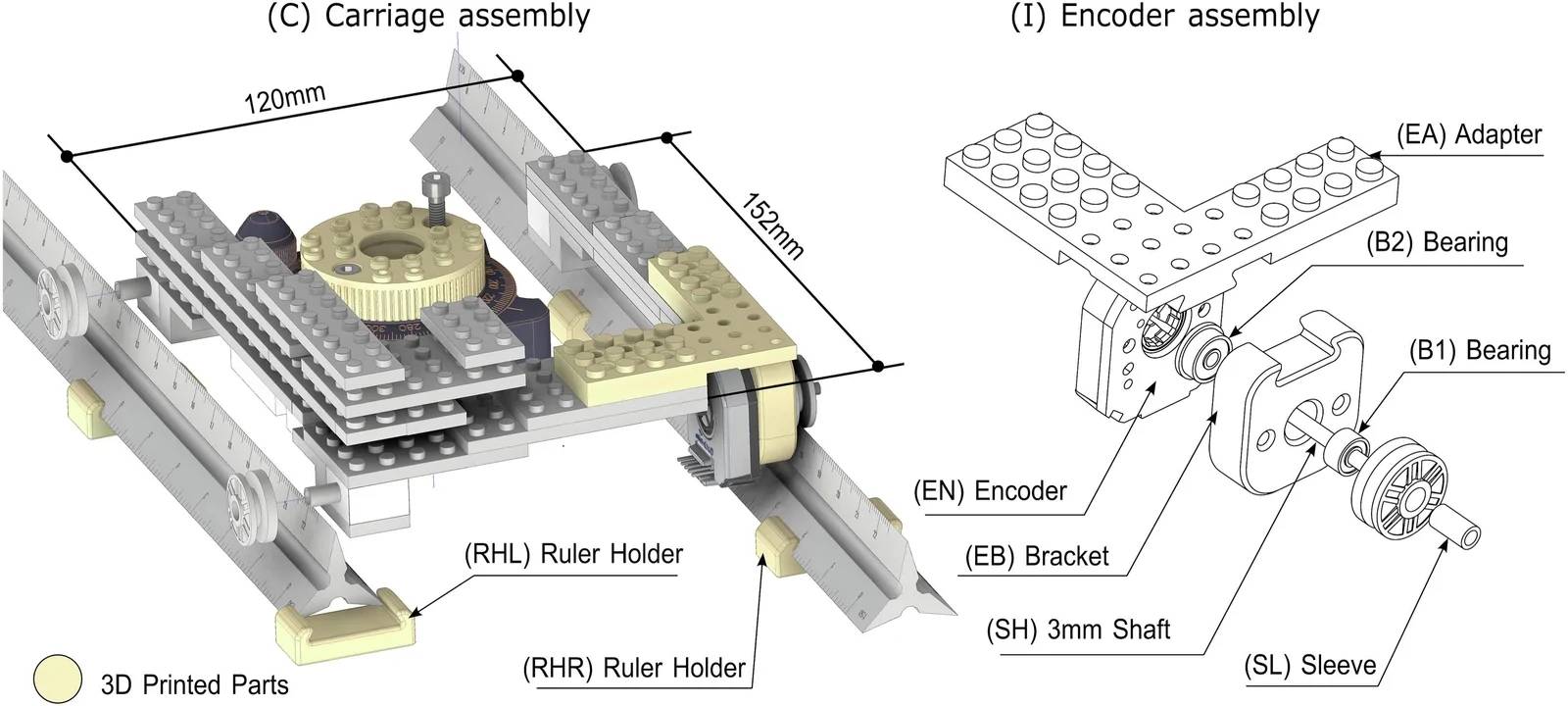
**Carriage and encoder mechanical assembly**. The U-shaped structure, built from standard LEGO bricks, is designed to support the optical scanning setup, allowing for clear rotation of the phantom without laser obstruction and supports the encoder assembly.
2. Encoder assembly: Mount the LEGO rim (part 13971) onto the sleeve (SL), then fit the rim–sleeve assembly onto the 3 mm shaft (SH), ensuring a proper interference fit at the joint. Install bearings B1 and B2 into the encoder bracket (EB). Seat the rim–sleeve–shaft assembly into the mounted bearings, verifying that no axial play is present prior to the encoder installation. A small amount of instant adhesive may be applied if necessary, but ensure that the rim rotates freely and smoothly. Finally, attach the modular incremental encoder (EN) to the shaft (SH) according to the instructions of the manufacturer [21]. The 3D‑printed component files are available in the project repository.
3. Wheels: Attach the wheels to the outer ends of the U-shaped carriage to enable smooth translation along the rails. Mount the triangular rulers (E) onto a LEGO (Ref: 3811) baseplate positioned on a flat surface. Using the provided 3D–printed adapters (RHL, RHR), secure the rulers firmly in place. Position the selected rotational setup at the midpoint between the rulers, as illustrated in Fig. 3 and 6.

### 5.2 Electronic assembly

The electronics focus on low-noise signal acquisition from the photodiode and reliable position readout from the encoder. Refer to the circuit schematic in Fig. 7 for specific wiring.

1. Photodiode circuit: Connect the photodiode to the amplification stage. We use an LMH6644 operational amplifier (OP) in a transimpedance amplifier (TIA) configuration with a feedback resistor $R_{f}=49k\Omega$, and a compensation capacitor $C_{1}=100\text{pF}$. We selected this combination to provide sufficient gain for low-intensity laser transmissions while maintaining a bandwidth capable of handling the scanning frequency.
2. Signal interfacing: Connect the output of the TIA to Pin 14 (A0) of the Teensy 4.0 microcontroller (UC). We chose the Teensy 4.0 for its high-speed ADC capabilities and 600 MHz clock, which allows for near-simultaneous sampling of position and intensity.
3. Encoder wiring: The AMT103 encoder signals (A and B channels) must be passed through a voltage divider (using 2 k Ω and 1 k Ω resistors, R1 to R4) to step down the 5 V logic to the 3.3 V tolerance of the Teensy pins (Pin 0 and Pin 1).
4. Laser wiring: Connect the laser module to a stable voltage source (PS). Verify the polarity of the power supply; the wall adapter specified in the Bill of Materials is equipped with a barrel plug, with the positive terminal at the center pin. The corresponding barrel jack (BJ) requires soldering to ensure proper electrical connection.
5. Power supply: Ensure a common ground between the Teensy, the Op-amp, and the encoder to prevent ground loops that could introduce periodic noise into the projection profiles.

**Fig. 7:**
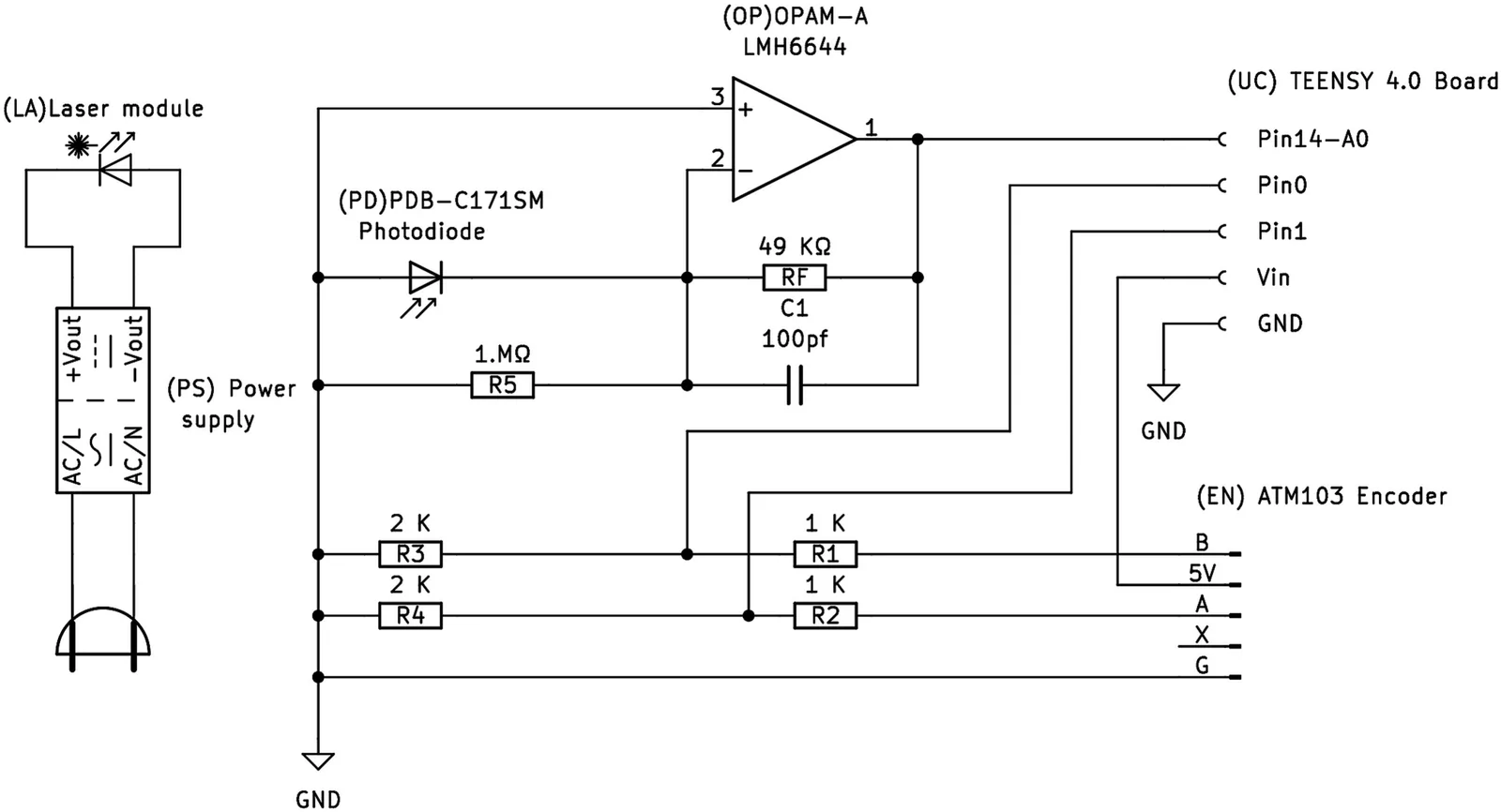
**Circuit schematic**. The diagram depicts the transimpedance amplifier stage for the photodiode, Teensy 4.0 microcontroller connections, the voltage divider for the encoder, laser module wiring, and the common ground distribution required for noise reduction.

### 5.3 Optical assembly

The optical train must be carefully aligned to ensure high‑quality projections and accurate results during Radon transform reconstruction. Once the entire system is on a flat surface, verify that all four wheels (LEGO rims, Ref. 13971) rotate smoothly along the rails. The assembled optical train should be positioned close to the wheels to minimize vertical misalignment during motion.

1. Laser assembly: Insert the laser module (LA) into the 3D‑printed support components as shown in Fig. 8. The “Laser Collect (LC)” and “Retaining Nut (RN)” mechanism provides secure adjustment of the laser module (LA) within the laser support (LS). Once aligned, place the complete assembly into the Laser‑to‑LEGO adapter (LAL).

**Fig. 8:**
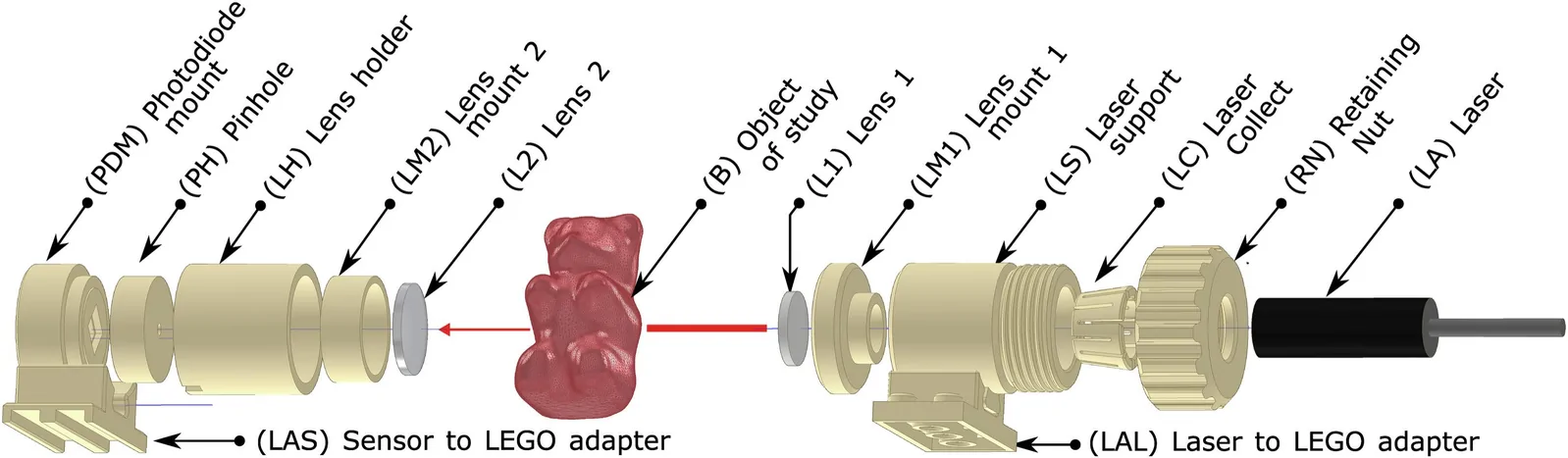
**Optical train**. The optical train configuration, featuring the laser diode within its mount, collimating lenses for beam shaping, and the detection assembly comprising a pinhole and photodiode, aligned for consistent beam transmission during the scan.
2. Sensor assembly: Solder two thin wires to the anode and cathode of the photodiode. The photodiode mount (PDM) provides clearance for routing these wires. Next, install the lens holder (LH) and secure it into the sensor adapter (LAS). This assembly must be positioned opposite the laser setup, with the lens holder aligned to the 3D‑printed 2 mm pinhole (PH). The pinhole serves as a cost‑effective alternative to a precision optical slit. To improve print quality, the 2 mm aperture can be rectified using a 2 mm screwdriver (e.g., PH00). This component effectively limits the field of view of the photodiode to the direct beam, thereby reducing the influence of scattered photons [7].
3. Alignment: Power the laser and adjust the mounts and lenses until the beam strikes the center of the photodiode active area, observe that the laser point keeps its position along the linear movement.
4. Laser focusing: Mount Lens 1 (L1) and Lens 2 (L2) into their respective 3D‑printed mount holders. Adjust their relative positions to focus the laser into a narrow beam at the desired plane. A properly focused beam is essential to approximate the parallel‑beam geometry required for standard filtered back‑projection algorithms [10].

Safety concerns.

● Laser safety: This device uses a Class 2 laser. Users should never look directly into the beam or its specular reflections. Use of laser safety goggles (specific to the wavelength of the laser) is mandatory during the alignment phase [7].
● Electrical safety: While the system operates at low voltages (5 V/3.3 V), ensure all connections are insulated to prevent short circuits that could damage the Teensy 4.0 microcontroller.

## 6 Operation instructions

The operation of the CT platform follows a sequential workflow involving manual mechanical operation for the projection profile generation, and software usage for digitalization and image reconstruction (see Fig. 9).

**Fig. 9:**
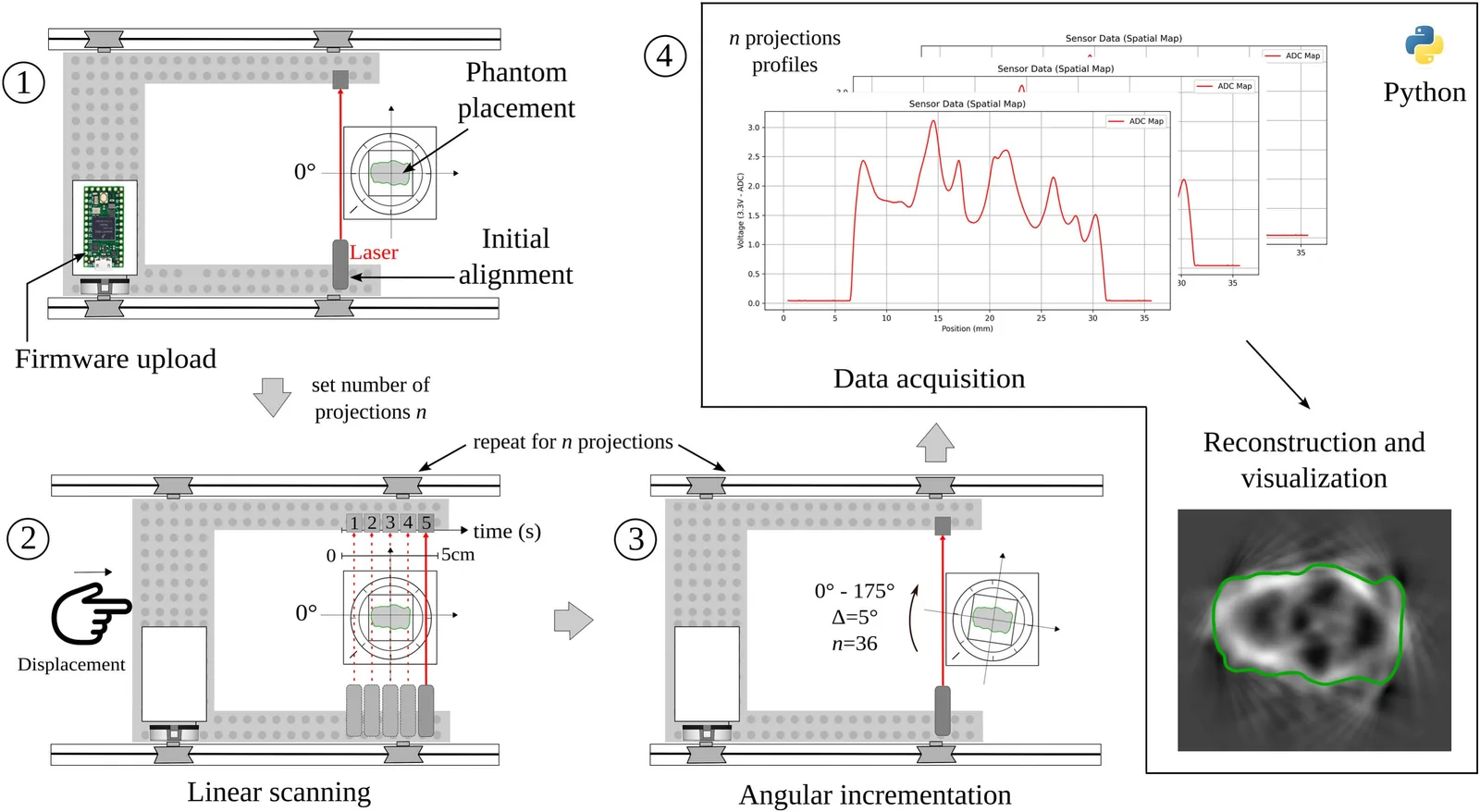
**Operational workflow of the optical CT platform.** The schematic outlines the sequential operational stages: 1. Uploading the firmware to the Teensy board (initial setup only), performing initial laser alignment, and centering the phantom on the rotational platform; 2. Manually conducting linear scans to generate projection profiles; 3. Increasing the rotation angle and repeating step 2 until the required number n of projections is reached; 4. Executing data acquisition, reconstruction, and image visualization via a JupyterLab notebook.

### 6.1 Projection profile generation

This stage involves the physical interaction between the user, the optical system, and the object under study to acquire raw attenuation data in the form of projection profiles.

1. Initial alignment: With the platform empty, ensure the laser beam and the optical sensor assembly are properly aligned. Confirm that the beam remains centered on the active area of the photodiode throughout the full travel length of the rail.
2. Phantom placement: Set the rotation platform to 0° and center the object of study. The object must remain fixed during scanning to avoid motion artifacts. At this stage, you can launch the JupyterLab Notebook to initiate the software-controlled process.
3. Linear scanning: Once prompted by the software, set the number of projections and manually slide the carriage along the rail for 5 cm at a constant velocity (approximately 1 cm/s). During this motion, the microcontroller records the linear position decoded from the encoder, while the ADC captures the intensity profile of the photodiode.
4. Angular incrementation: After completing a linear pass, manually rotate the platform by the specified angular increment (e.g., 5° for 36 projections, rotating from 0° to 170°). Repeat the process until all projections are acquired. The software will provide a notification after each cycle and upon completion of the full sequence.

### 6.2 Software usage and image reconstruction

The software workflow facilitates the transition from raw projection profile data to the visualization of the reconstructed image. The process is executed in a Python environment within JupyterLab (the script is available in the file repository).

1. Firmware upload: Flash the Teensy 4.0 with the provided.ino acquisition script. This script synchronizes the ADC readings from the photodiode with the interrupts generated by the encoder.
2. Data acquisition: Launch the JupyterLab Notebook. The software will prompt for the number of projections and then guide the user through the remaining sequences. As the projections are acquired, the software aggregates individual linear scans into a 2D matrix known as a sinogram, where the horizontal axis corresponds to linear displacement and the vertical axis to rotation angle. The sinogram matrix can be exported as a CSV file for further analysis.
3. Reconstruction: Once all projection scans are complete, the raw data is processed. The reconstruction script applies a Filtered Back-Projection algorithm to generate the image. Users can select different filters in the “Reconstruction” tab to observe how frequency-domain filtering affects image sharpness and noise.
4. Visualization: The resulting 2D array is mapped to a grayscale image, representing the internal structure and linear attenuation coefficients of the phantom. Users can then analyze the geometric fidelity and density differentiation of the reconstructed object.

To test and understand the image reconstruction process without an operational hardware platform, the Python algorithm allows users to upload previously obtained sinogram files in CSV format to perform the respective image reconstruction. Two CSV sinogram files are available in the project repository for direct use in the algorithm.

## 7 Validation

### 7.1 Technical validation

The fully assembled optical CT educational platform, characterized by its compact size and cost-effective implementation, operated consistently throughout the experimental trials, as quantified by the detector SNR and the end-to-end reproducibility analysis reported below. Its modular architecture serves as a robust platform for illustrating fundamental tomographic principles through tangible hardware-software interaction. The following subsections detail the validation of the system using diverse phantoms to evaluate its response to varying optical properties.

Three reconstruction methods implemented in scikit-image were used, as specified in each figure caption: filtered back-projection (FBP; inverse Radon transform with a Hamming filter), the simultaneous algebraic reconstruction technique (SART) with Gaussian filtering between iterations (five iterations, σ = 0.30), and SART with total-variation (TV) regularization for sparse-view data. Unless otherwise stated, scans comprised 36 projections over 0–180°. FBP is the default method of the practice, whereas the iterative variants, including TV regularization, are optional steps used to illustrate artifact reduction and are not part of the standard pipeline. Their parameters, as well as the center-of-rotation correction described below, were selected empirically by visual inspection of artifact reduction; all of them are exposed in the notebook so that students can explore their effect.

*Phantoms and density differentiation*

To evaluate the sensitivity of the system to internal density variations, a gummy bear was used as a soft-tissue-mimicking phantom. The initial reconstruction (Fig. 10a) successfully resolved the general morphology of the object, capturing subtle changes in attenuation across its semi-translucent structure. Further validation was conducted by introducing a high-opacity inclusion, a metal screw, within the body of the phantom (Fig. 10b). The resulting tomographic slice clearly showed the presence of the screw, manifesting as a sharp increase in

**Fig. 10:**
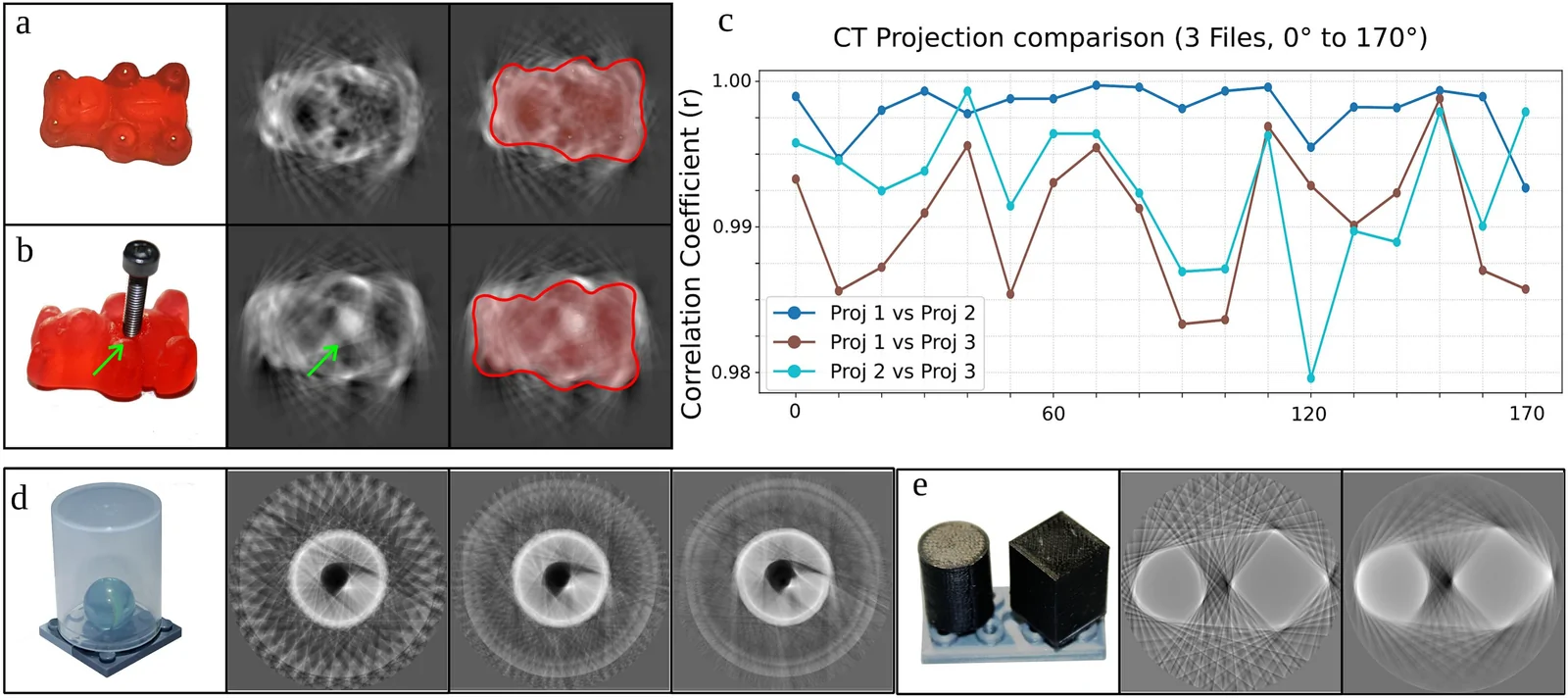
**Experimental tomographic reconstructions obtained with the optical CT educational platform.** (a) Gummy bear phantom: photograph (left), sparse-view reconstruction from 36 projections with SART and total-variation (TV) regularization (center), and the same reconstruction with the phantom contour superimposed (right). (b) Gummy bear phantom with a metal screw inclusion (green arrow), shown as in (a). (c) Reproducibility of raw projection profiles: Pearson correlation coefficient between corresponding projections (0–170°, 10° steps) of three independent acquisitions: Proj. 1, Proj. 2, and Proj. 3 (see Table 4). (d) Glass marble inside a cylindrical plastic cap: photograph and FBP (inverse Radon) reconstructions with 18, 36, and 90 projections (left to right). (e) 3D-printed cylindrical and cubic phantoms: photograph (left), FBP reconstruction (center), and SART reconstruction with Gaussian filtering (σ = 0.30; right), both from 36 projections. (For interpretation of the references to colour in this figure legend, the reader is referred to the web version of this article.)

*Translucent media and internal refraction*

Validation was extended to translucent objects, specifically a spherical glass marble inside a cylindrical plastic cap (Fig. 10d), to observe the capacity of the system for capturing internal structures in media where light travel is not strictly linear [22]. The reconstruction successfully identified internal refractive features, illustrating how visible light interacts with varying indices of refraction [23]. This trial underscores a key educational takeaway: while conventional CT assumes straight-line ray paths, optical CT must contend with complex light behavior, such as internal refraction and scattering, which are preserved in the final image provided by the system [24], [25]. Because the platform measures the light that reaches a pinhole-limited detector, the reconstructed values represent an apparent (extinction) attenuation that combines absorption, scattering, and refraction-induced deflection, rather than the linear attenuation coefficient of the glass. In the FBP reconstructions of Fig. 10d, the bright annulus at the boundary of the marble arises mainly from rays strongly deflected at the curved air–glass interface, which miss the detector and are therefore recorded as attenuated, whereas rays crossing the center of the sphere are only weakly deviated and appear with low apparent attenuation; the faint outer ring corresponds to the wall of the plastic cap. Increasing the number of projections (18, 36, and 90) reduces the streak pattern without altering these refraction-dominated features (see also Fig. 11 and Table 5). These images should therefore not be interpreted as X-ray attenuation maps. Nevertheless, the physical deviations introduced by visible light give rise to artifacts that are qualitative analogues of those encountered in clinical CT: refraction-induced edge artifacts recall the edge and cupping effects of beam hardening, doubled contours caused by rotation-axis misalignment reproduce the effects of gantry misalignment and patient motion, and sparse-angle streaking corresponds to the view-aliasing streaks of sparse-view, dose-reduced acquisitions. Observing, explaining, and correcting these artifacts on a safe, non-ionizing platform is a central part of its pedagogical value.

**Fig. 11:**
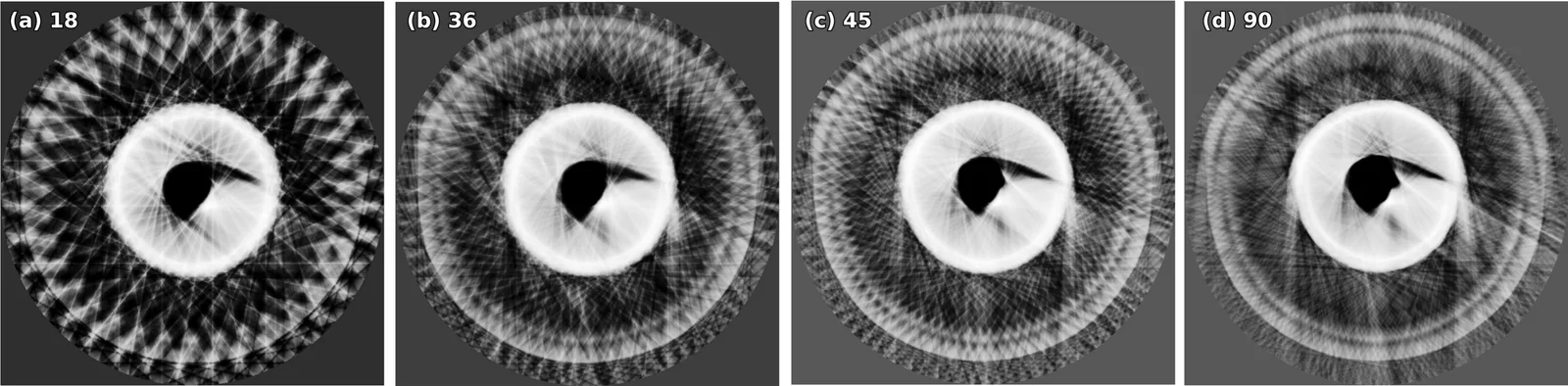
**Effect of angular sampling density on the reconstruction of the marble–cap phantom** (16-mm glass marble inside a 32-mm plastic cap): (a) 18, (b) 36, (c) 45, and (d) 90 projections, reconstructed with SART (five iterations, Hamming window) and Gaussian filtering (σ = 0.30). Images are contrast-enhanced for visualization.

*Geometric fidelity of opaque phantoms*.

The final validation stage employed 3D-printed geometric shapes (cylinder ∅=10mm and cube l=10mm) to assess spatial accuracy and symmetry (Fig. 10e). These opaque objects provided a high-contrast boundary that validated the ability of the system to preserve general symmetry and produce faithful approximations of external object dimensions. The reconstructed profiles showed dimensions consistent with those of the physical models, indicating that the acquisition geometry and the reconstruction are properly matched; this agreement is quantified below by the relative area error. To evaluate the performance of the SART reconstruction with Gaussian filtering, a quantitative comparison was conducted against the ground truth geometry of the 3D-printed phantoms [7]. The accuracy was measured using the Relative Area Error (RAE) and Circularity Index as indicators of area conservation and shape fidelity, respectively. Fig. 10e compares the raw FBP reconstruction of these phantoms, dominated by the streak artifacts typical of sparse-view sampling, with the SART reconstruction with Gaussian filtering, in which the streaks are attenuated and the object interiors appear more homogeneous.

Considering a scanning process of 36 projections in total, the reconstruction maintained high spatial fidelity for the cylindrical region, with a marginal RAE of 0.94 %. Conversely, the cubic phantom exhibited a significant RAE of 32.19%. This discrepancy is largely attributable to the “corner-rounding” effect and the integration of tangential streak artifacts into the segmented boundary [26]. The Gaussian post-filter, while effective at suppressing noise and streaks, also smooths sharp discontinuities under sparse-view conditions, contributing to the observed expansion of the cubic edges [7]. These results suggest that while this reconstruction is robust for curvilinear geometries, additional constraint terms (e.g., edge-preserving penalties) may be required for high-accuracy reconstruction of angular structures in limited-data scenarios.

The performance of the proposed modular optical CT platform is constrained by intentional design choices that balance pedagogical feasibility with hardware simplicity. The reliance on sparse angular sampling, restricted to 36 projections per scan, inherently fails to satisfy the Nyquist-Shannon sampling criterion, resulting in characteristic streaking and blurring artifacts that degrade image resolution compared to industrial systems [23], [27]. Furthermore, the visible-light laser source, while safe and cost-effective, possesses limited penetration power compared to ionizing X-ray radiation. This limitation restricts the utility of the system primarily to small, semitranslucent phantoms, as the light beam undergoes significant attenuation in opaque or high-density media, rendering internal structure resolution infeasible for such objects [22]. Additionally, standard Radon transform-based reconstruction algorithms rely on a linear ray-path assumption that is violated in practice. Complex optical phenomena (including reflection, refraction, and diffraction at material interfaces) introduce spatial distortions that compromise reconstruction fidelity [24]. Addressing these non-linear effects would necessitate advanced diffraction-based algorithms or index-matching protocols, thereby increasing the cost of the system and technical complexity beyond its current educational scope.

Despite these technical constraints, the trade-off between hardware simplicity and image quality remains a defining and valuable characteristic of the platform. While the physical limitations of the laser and the sparse sampling prevent the device from being used in medical or industrial diagnostic applications, they provide a tangible learning opportunity for students. By directly observing these artifacts and the physical consequences of low-energy radiation, learners gain a practical understanding of the real-world compromises required between acquisition time, hardware costs, and the mathematical integrity of tomographic imaging [22], [27]. This modular approach effectively bridges the gap between theoretical simulation and high-end industrial systems through accessible, hands-on experimentation.

*Detector signal-to-noise ratio*.

The signal-to-noise ratio (SNR) of the detection chain was estimated directly from the raw projection profiles as SNR = (*S_block_* − *S_free_*)/*σ_free_*, where *S_free_* and *σ_free_* are the mean and standard deviation of the digitized signal in the unobstructed-beam regions at both ends of each profile (50 samples per side), and *S_block_* is the mean signal when the beam is fully blocked by the phantom. Across five independent acquisitions of the gummy bear phantom (18, 36, 45, and 90 projections, plus the 90-projection reference dataset), the baseline noise ranged from 1.3 to 1.7 mV (approximately two least-significant bits of the 12-bit ADC), yielding an SNR of 2257 ± 282, i.e., 67.0 ± 1.1 dB (range 65.9–68.2 dB). This value characterizes the photodiode and signal-conditioning stage; it should not be confused with an image-domain SNR, which in the reconstructed slices is further limited by sparse angular sampling and refraction artifacts.

*Angular positioning and end-to-end reproducibility*.

Because the rotational stage is operated manually, the angular position of each projection is set by the operator using the 2° graduation markings of the RSP-1 T mount, which also provides a fine-adjustment knob with a sensitivity of 4 arc-min and a locking screw [17]. As the platform does not include an independent calibrated angular reference, no standalone numerical accuracy or repeatability was assigned to the manual stage itself; instead, positioning and acquisition reproducibility were evaluated experimentally at the system level.

The same gummy bear phantom was scanned in three independent acquisitions over 0–180° (Proj. 1, Proj. 2, and Proj. 3). Since these datasets share 18 nominal angles at 10° intervals (0–170°), the corresponding raw projection profiles (690 samples at ≈0.051 mm) were compared pairwise using the Pearson correlation coefficient (*r*), the relative difference in integrated intensity, and the lateral shift between profiles estimated from the peak of their cross-correlation (Fig. 10c and Table 4). The Proj. 1 and Proj. 2 acquisitions were highly consistent (mean *r* = 0.998, range 0.993–1.000), with a mean relative integrated-intensity difference of 1.0 % (−0.7 % to 3.4 %) and a mean lateral shift of 0.01 ± 0.02 mm, i.e., below one sampling step (≈0.051 mm). Comparisons involving the Proj. 3 acquisition also showed strong correlation (mean *r* ≥ 0.991), but revealed a systematic offset, with integrated intensities 1.5–6.3 % lower and profiles shifted by approximately 0.1 mm (two samples). Because the profile shapes were preserved, this offset most likely reflects small between-session differences in phantom placement or illumination rather than a failure of the encoder-based synchronization.

**Table 4:** End-to-end reproducibility of raw projection profiles acquired at 18 common angles (0–170°, 10° steps) in three independent acquisitions of the same phantom.

**Compared acquisitions**	**Pearson r, mean (range)**	**Relative integrated-intensity difference, mean (range)**	**Lateral shift (mm), mean ± SD (max. |shift|)**
Proj. 1 vs. Proj. 2	0.998 (0.993–1.000)	1.0 % (−0.7 % to 3.4 %)	0.01 ± 0.02 (0.06)
Proj. 1 vs. Proj. 3	0.991 (0.983–0.999)	3.9 % (1.5 % to 6.3 %)	0.11 ± 0.07 (0.25)
Proj. 2 vs. Proj. 3	0.993 (0.980–0.999)	2.9 % (1.8 % to 5.6 %)	0.08 ± 0.07 (0.22)

Since these metrics were computed on raw projections, they intrinsically incorporate the variability of the complete acquisition chain, including manual angular positioning, linear translation, encoder-based synchronization, optical interaction with the phantom, signal conditioning, and analog-to-digital conversion. They therefore characterize the end-to-end reproducibility of the acquisition process rather than the isolated mechanical repeatability of individual components.

Phantom centering relative to the rotation axis is the main residual geometric error of the manual platform, since an offset between the rotation axis and the center of the scan produces doubled contours and blurring. This offset was compensated in software by shifting the sinogram by 10 samples (≈0.5 mm), a value chosen empirically: larger shifts further improved the roundness of the cylindrical phantom but degraded the squareness of the cubic phantom, indicating over-correction. Together with the 2° graduations of the rotation mount and the sub-sample lateral reproducibility reported above, this indicates that mechanical errors remain small relative to the effective spatial resolution while still being observable, so that students can vary the center-of-rotation correction and directly see its effect on the reconstruction. A dedicated calibration of angular and centering errors, for example with a motorized stage and a thin centered pin phantom, is planned for future versions of the platform.

Relative difference computed per angle as (Σ*S_a_* − Σ*S_b_*)/Σ*S_a_* × 100, where *a* is the first acquisition of each pair; lateral shift estimated from the cross-correlation peak with parabolic sub-sample interpolation.

*Effect of the number of projections*.

To quantify the effect of angular sampling density, a phantom of known geometry, a 16-mm glass marble centered inside a plastic cap (32-mm outer and 28.6-mm inner diameter), was reconstructed from 18, 36, 45, and 90 projections over 0–180° (Fig. 11) using SART (five iterations, Hamming window) combined with Gaussian filtering (σ = 0.30). The images were segmented by four-class multi-Otsu thresholding, and the spatial scale (≈0.051 mm per pixel, consistent with the native sampling of the platform) was calibrated with the known outer diameter of the cap; hence, the cap served only for calibration, and the marble provided an independent geometric reference. For each reconstruction, the enclosing-circle diameter, relative area error (RAE), and circularity (4π*A*/*P*^2^, where *A* and *P* are the segmented area and perimeter) of the marble were computed, and the global reconstruction error was quantified with respect to the 90-projection reconstruction using the normalized root-mean-square error (NRMSE) and the structural similarity index (SSIM) (Table 5).

**Table 5:** Effect of the number of projections on the reconstruction of the marble–cap phantom.

**Projections**	**Marble diameter, mm (error)**	**Marble RAE**	**Marble circularity**	**NRMSE vs. 90 proj.**	**SSIM vs. 90 proj.**
18	15.98 (0.14 %)	4.37 %	0.897	0.047	0.960
36	15.89 (0.71 %)	5.62 %	0.899	0.040	0.969
45	15.96 (0.28 %)	6.12 %	0.895	0.020	0.988
90	16.09 (0.54 %)	7.28 %	0.865	Reference	Reference

Increasing the number of projections progressively reduced streak artifacts and the deviation from the 90-projection reconstruction (NRMSE from 0.047 to 0.020; SSIM from 0.960 to 0.988). The marble diameter remained within 0.7 % of its nominal value at all sampling densities, whereas its RAE (4.4–7.3 %) and circularity (0.87–0.90) did not improve systematically with angular sampling. This indicates that, from 18 projections onwards, geometric fidelity is limited mainly by refraction at the curved glass interfaces, which produces irregular segmented boundaries, whereas angular sampling chiefly determines the severity of the streak artifacts that students can directly observe.

Diameter: enclosing-circle diameter of the segmented marble (error relative to the nominal 16 mm). Spatial scale calibrated with the 32-mm outer diameter of the cap.

*Comparison with other low-cost educational platforms*.

Table 6 compares the proposed platform with other low-cost educational optical CT systems in terms of mechanical design, position–intensity synchronization, reconstruction, spatial resolution, signal-to-noise ratio (SNR), cost, and open-source resources. Among the systems that report hardware cost, the proposed platform has the lowest upper bound (USD 408–631, Table 2), compared with approximately EUR 150–1000 for kitchen-based light tomography [14] and EUR 300–1500 for the educational scanner of Prochazka et al. [7], and it is roughly two orders of magnitude less expensive than commercial educational CT kits (≈EUR 39,000, as reported in [7]). It is also the only system in the comparison that combines hardware-level position–intensity synchronization through a dedicated encoder with an open release of CAD files, firmware, software, and datasets. Conversely, manual rotation and a single-point detector limit acquisition speed and automation; this trade-off was intentionally accepted so that each stage of the acquisition chain remains visible to and operable by the student. Regarding spatial sampling, the native pixel of the proposed platform (≈0.0026 mm^2^) is considerably finer than the 2.67 mm^2^ per pixel reported in [7], although in both systems the effective resolution is ultimately limited by the width of the optical beam.

**Table 6:** Comparison of low-cost educational optical computed tomography platforms.

**Platform**	**Mechanical design and acquisition**	**Position–intensity synchronization**	**Reconstruction (algorithm / software)**	**Spatial resolution**	**SNR**	**Approximate cost**	**Open-source resources**
**This work**	LEGO® chassis and 3D-printed parts; manually rotated platform; linear carriage on rails; 635 nm diode laser and single photodiode	Capacitive incremental encoder (AMT103); continuous position–intensity synchronization during translation	FBP / inverse Radon transform; Python (scikit-image), JupyterLab	≈0.051 × 0.051 mm pixel (≈0.0026 mm^2^/pixel; reconstruction grid); RAE 0.94 % (cylinder) and 32.19 % (cube) at 36 projections	≈67 dB (65.9–68.2)	USD 408–631	CAD (IPT/IAM/STEP/STL), firmware (.ino), Jupyter notebook, CSV datasets; GNU-GPL v3.0 / MIT / CC BY 4.0
Prochazka et al. [7], 2025	Replica of a third-generation CT (motorized gantry; bed with height and translation motion); 259 3D-printed parts; Xilinx Spartan-6 FPGA; class IIIa 650 nm line laser and 64-phototransistor array	FPGA control of stepper motors; binary detection (beam blocked/unblocked); no dedicated encoder	FBP / inverse Radon transform; MATLAB	2.67 mm^2^/pixel; 44 × 44 px final image	ND	EUR 300–1500	No open design repository indicated
Larsson et al. [14], 2023	“Kitchen-based” DIY kit; fixed visible-light source and digital camera	ND	Open reconstruction software (published toolkit)	ND	ND	EUR 150–1000^a^	Open-source DIY toolkit
Fong-on et al. [28], 2018	Four Raspberry Pi cameras on a rotating gantry acquiring cone-beam projections; no scanning light beam	Multi-camera capture; synchronization not detailed	ND	256 × 256 px (cropped)	ND	ND	ND
Narkbuakaew et al. [29], 2005	Single camera on dual linear rails; combined translation and rotation producing a spiral (helical) trajectory	ND	ND	≈256 px (pitch-dependent)	ND	ND	ND

ND: not determined or not reported in the cited source (convention adopted from Table 1. in [7]); FBP: filtered back-projection; RAE: relative area error; SNR: signal-to-noise ratio (for this work, measured at the detector level; see Section 7.1). ^a^ Cost as reported in [7].

### 7.2 Pedagogical validation: Pilot study with students

To obtain preliminary evidence of the pedagogical feasibility of the platform, a single-group pre–post pilot study was conducted with 16 undergraduate bioengineering students who had previously completed the Medical Imaging in Bioengineering course. The pilot was designed to test the feasibility of the practice and of the evaluation instruments, not as a controlled educational trial. Each session comprised a 10-min introductory talk and a 15-min guided practice in which students acquired projections of a phantom by manually rotating the platform to the angles indicated by the software, observed the sinogram being built projection by projection, and reconstructed the cross-section by FBP. Conceptual understanding was assessed with a five-item multiple-choice questionnaire (sinogram, reconstruction algorithm, sparse angular sampling, Radon transform, and CT workflow) completed individually immediately before and after the practice. Perception of use was assessed afterwards with a 10-item, 5-point Likert survey covering usability, perceived conceptual support, and motivation, followed by four open-ended questions. Both instruments were anonymous. Given the small sample and the ordinal nature of the data, paired scores were compared with the exact Wilcoxon signed-rank test, reporting the matched-pairs rank-biserial correlation (*r*) and Hake’s class-average normalized gain, 〈*g*〉 = (〈post〉 − 〈pre〉)/(5 − 〈pre〉); the mean perception score was compared with the neutral midpoint (3) using an exact one-sample Wilcoxon test.

Fourteen students returned the perception survey and ten returned complete paired questionnaires. Baseline performance was already high (3.8 ± 0.9 out of 5; 76 % correct answers), as expected for students who had completed the course. After the practice, the mean score increased to 4.3 ± 0.8 (86 %), and the number of perfect scores rose from two to five (Fig. 12a). The mean change (+0.5 points; bootstrap 95 % CI − 0.1 to 1.2) corresponded to 〈*g*〉 = 0.42 and *r* = 0.67, although it did not reach statistical significance with this sample size. The largest item-level gains were observed for the sinogram and reconstruction-algorithm questions (from 70 % to 90 % correct each), whereas the Radon-transform item remained at 50 % (Fig. 12b). Perception was uniformly positive (Fig. 12c): 95 % of all ratings were “strongly agree”, none were negative, and the mean score per participant (4.94 ± 0.08) was significantly above the neutral midpoint (*p* < 0.001). All students rated the platform as easy and safe to operate and would recommend its use in the course. In the open-ended answers, 11 of the 14 students (79 %) identified the sinogram as the concept the device helped them understand, frequently attributing this to watching the measured profile change with each projection. The most frequent suggestions were motorized rotation (6/14) and shielding from ambient light (4/14). These results constitute preliminary evidence of feasibility and acceptance rather than of learning effectiveness. The pilot was limited by its small sample, a ceiling effect among participants who had already taken the course, and an immediate post-test with identical items.

**Fig. 12:**
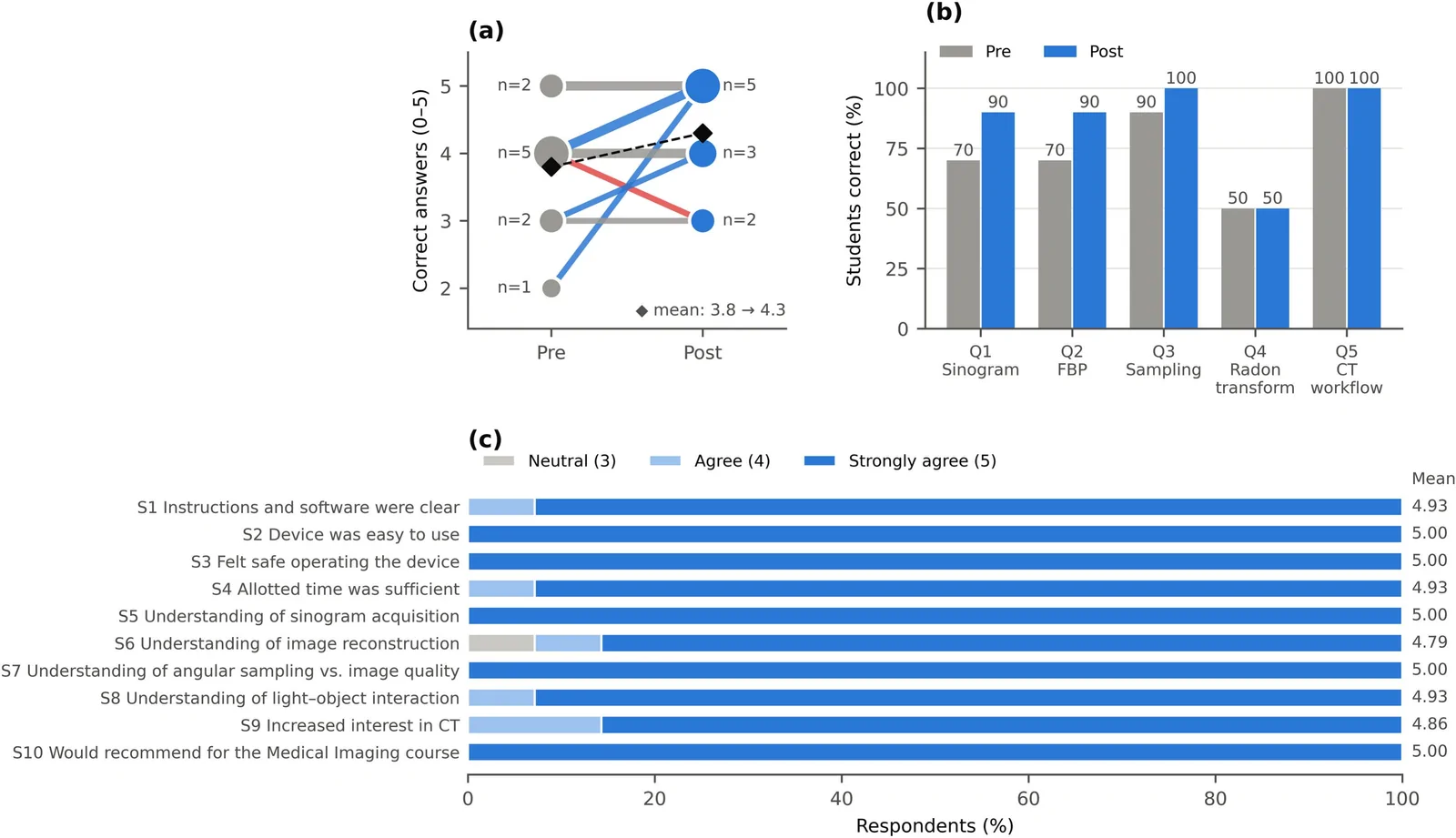
**Results of the pilot study with students.** (a) Paired pre/post scores in the five-item conceptual questionnaire (n = 10); line width and marker size are proportional to the number of students, blue lines indicate improvement, gray lines no change, and the red line a decrease; diamonds indicate group means. (b) Percentage of correct answers per item before and after the practice. (c) Distribution of responses to the five-point Likert perception survey (n = 14; no responses of 1 or 2 were recorded); values on the right are item means. (For interpretation of the references to colour in this figure legend, the reader is referred to the web version of this article.)

In addition, two instructors in biomedical instrumentation, with 8 and 10 years of teaching experience, completed the same perception survey after using the platform. They rated all items at 4 or 5 (mean scores of 4.9 and 4.7) and highlighted the link between sinogram acquisition and the Nyquist sampling criterion as key to understanding image reconstruction. In agreement with the students, they suggested automating the z-axis of the device; they also proposed integrating clinical case scenarios into the practice and developing a web-based version of the software.

### 7.3 Conclusion

This project introduces a modular, low-cost optical CT platform designed to bridge the gap between theoretical tomography and clinical-grade imaging equipment. By leveraging accessible components such as building blocks and microcontrollers, the system provides a tangible learning environment that facilitates education in these complex topics. Unlike high-end clinical scanners, which use ionizing radiation and complex acquisition geometries, this platform intentionally operates within hardware constraints (specifically limited angular sampling and visible-light optics). While these design choices result in characteristic image artifacts, they serve a critical pedagogical function: enabling students to directly observe the mathematical and physical consequences of sparse-view reconstruction and refractive non-linearities. Consequently, the hardware does not merely emulate CT principles; it provides a unique laboratory tool that exposes the inherent trade-offs between hardware costs, acquisition speed, and image fidelity, effectively preparing learners for the complexities of modern diagnostic imaging systems.

## Ethics statement

The pilot study received technical and ethical approval from the Technical Research Committee of the Faculty of Engineering, Pontificia Universidad Javeriana (31-08-2026 000 − ID). All participants provided written and signed informed consent, and no personally identifiable information was collected with the questionnaires.

## Data availability

All design files (CAD.IPT/.IAM/.STEP/.STL, 3D printing guide), firmware (.ino), reconstruction software (JupyterLab notebook), and representative sinogram datasets (.csv) that support this study are openly available on Zenodo at https://doi.org/10.5281/zenodo.23484086. Hardware design files are released under the GNU General Public License v3.0, software under the MIT License, and documentation under CC BY 4.0.

## Declaration of generative AI and AI-assisted technologies in the manuscript preparation process

During the preparation of this work, the authors used Jenni.ai in order to perform English language editing and style refinement. After using this service, the authors reviewed and edited the content as needed and take full responsibility for the content of the published article.

## Funding

This research received no specific grant from any funding agency in the public, commercial, or not-for-profit sectors.

## Declaration of competing interest

The authors declare that they have no known competing financial interests or personal relationships that could have appeared to influence the work reported in this paper.
